# Metabarcoding Reveals the Dietary Patterns of Bats Within a Unique European Habitat, the Pasture‐Dominated Landscapes of Ireland

**DOI:** 10.1002/ece3.72310

**Published:** 2025-10-28

**Authors:** G. Hurpy, T. Aughney, I. Skujina, N. Roche, E. C. Teeling

**Affiliations:** ^1^ School of Biology and Environmental Science University College Dublin, Belfield Dublin 4 Ireland; ^2^ Bat Eco Services Limited Virginia Co. Cavan Ireland; ^3^ Genome Stability Laboratory, School of Biological and Chemical Sciences University of Galway Galway Ireland; ^4^ Bat Conservation Ireland Dublin 7 Ireland

**Keywords:** Chiroptera, diet, faeces, *Pipistrellus*, *Plecotus*, trophic‐niche

## Abstract

Considered as keystone predators, insectivorous bats play essential roles in maintaining the functioning of ecosystems. Investigating how bat species' diets vary across landscapes is crucial for understanding bat ecology and their role in ecosystem health. Here, we characterised the predator–prey interactions of two common bat species with different foraging strategies, the Brown long‐eared bat ( *Plecotus auritus* ) and Soprano pipistrelle ( *Pipistrellus pygmaeus* ), across the unique pastureland‐dominated landscape of Ireland. Over 3 years (2021–2023), faecal samples (*n* = 4627 in total) were collected annually at three time points (gestation, lactation, post‐lactation) from 12 maternity roosts and analysed using metabarcoding and next‐generation sequencing. Both bat species showed broad diet diversity, with 392 and 350 arthropod species identified for the Brown long‐eared bat and Soprano pipistrelle, respectively, primarily Lepidoptera and Diptera. The Brown long‐eared bat exhibited a generalist diet, demonstrating dietary flexibility. Lepidoptera interactions were more frequent overall (62%) compared to Diptera (31%), but interactions with Diptera species increased markedly at one specific roost, suggesting that this species can opportunistically feed on available prey species. In contrast, the Soprano pipistrelle exhibited a more specialised diet, with 83% consisting of Diptera species. Both spatial and temporal factors significantly influenced dietary richness and composition in both species. Surrounding land cover, in particular, played an important role in shaping diet composition. Our findings suggest that the Brown long‐eared bat exhibits a broad foraging strategy, acting as a generalist with a preference for Lepidoptera, while the Soprano pipistrelle shows a consistent reliance on Diptera. Our study provides new insights into bat diet variation in pastureland landscapes, contributing to the understanding of their ecological role.

## Graphical abstract

We investigated the diets of two common insectivorous bat species, *Plecotus auritus* and *Pipistrellus pygmaeus* , across the pastureland‐dominated landscape of Ireland. Using metabarcoding and next‐generation sequencing, we analysed 4627 faecal samples collected over three years from twelve maternity roosts during gestation, lactation, and post‐lactation periods. Our findings suggest that *P. auritus* exhibits a broad foraging strategy, acting as a generalist with a preference for Lepidoptera, while *P. pygmaeus* shows a consistent reliance on Diptera. Both diets were strongly influenced by spatial and temporal factors.

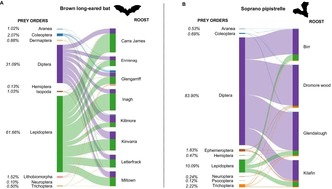

## 1 Introduction

The landscape of Ireland is clearly distinct within Europe, with grasslands covering 57%–61% of the land, the highest proportion in Europe compared to an average of 17.4% across Europe (Haughey 2021; Eurostat 2024). Forest cover remains low (11%–14%) relative to the EU average (41.1%), and cropland accounts for just 5%–9.5%, ranking Ireland among the lowest in Europe alongside Sweden and Finland (Eurostat 2024). This unique Irish landscape structure, dominated by grasslands, may lead to differences in Irish bat diets compared to more forested or intensive agricultural regions of Europe. Previous studies have shown that habitat characteristics strongly influence bat dietary composition (Mata et al. 2021; Tournayre et al. 2021; O'Rourke et al. 2022; Perea et al. 2025). Additionally, previous studies have suggested that arthropod species diversity may be relatively low in Ireland (McCarthy 1986; Ferriss et al. 2009; Nelson et al. 2011; Kelly‐Quinn and Regan 2012; Harrison 2014; Feeley et al. 2020), which could contribute to lower prey richness in Irish bat diets compared to other European regions.

Insectivorous bats feed on a broad diversity of arthropods and contribute to well‐functioning ecosystems (Kunz et al. 2011; Ghanem and Voigt 2012; Lacher et al. 2019; Ramírez‐Fráncel et al. 2022). Numerous studies have highlighted the importance of ecosystem services provided by insectivorous bats to agriculture, agroforestry, and even human health (Aizpurua et al. 2018; Puig‐Montserrat et al. 2020; Tiede et al. 2020; Baroja et al. 2021; Charbonnier et al. 2021; Montauban et al. 2021; Da Silva et al. 2023; Hunninck et al. 2022; Frank 2024). However, the majority of these studies focused on the services provided in relation to a single crop or one specific type of landscape, such as vineyards or forests, and there is little research examining the potential ecosystem services provided by bats in pastureland (Tuneu‐Corral et al. 2023). To inform national policy effectively, it is crucial to investigate the ecosystem services provided by bats on a broader scale (Braat and De Groot 2012; Guerry et al. 2015). This requires a comprehensive understanding of bat diets across diverse and representative landscapes within countries.

To date, only five studies have investigated the diet of bats in Ireland, focusing on a limited number of species and sites (McAney and Fairley 1989; Shiel et al. 1991, 1998; Flavin et al. 2001; Curran et al. 2022). Significant variations in the diet of the Lesser horseshoe bat ( *Rhinolophus hipposideros* ) (McAney and Fairley 1989) and Leisler's bat ( *Nyctalus leisleri* ) (Shiel et al. 1998) were observed across roosts and seasons, demonstrating how environmental changes affect prey availability. Shiel et al. (1998) found that the diet of Leisler's bats in Ireland showed lower richness compared to roosts in England and Germany, indicating that Irish land cover may significantly impact bat dietary diversity. These findings highlight that both landscape and temporal factors significantly influence bat diets. However, the limited studies underscore a considerable gap in understanding Irish bat feeding ecology. Comprehensive research on bat diets across diverse habitats and timeframes is essential to understanding their ecological roles and ecosystem services, particularly as Ireland's unique landscape may further shape the dietary habits of its local bat populations.

Among the nine bat species found in Ireland, the Soprano pipistrelle ( *Pipistrellus pygmaeus* ) is the second most abundant bat species (Roche et al. 2014). Although the Brown long‐eared bat ( *Plecotus auritus* ) is less abundant, it is also widely distributed and regarded as common (Roche et al. 2014). Despite their prevalence, little is known about the diets of these two species in Ireland, presenting a knowledge gap in the understanding of their diet. The stability and accessibility of their maternity roosts, which are typically found in buildings from May to August, provide an ideal opportunity for non‐invasive faecal sampling methods, making these species particularly suitable for diet studies. Furthermore, the distinct foraging behaviours of these species offer valuable insights into their ecological roles: the Brown long‐eared bat is a gleaning species that forages within and on vegetation, while the Soprano pipistrelle uses aerial hawking to capture prey at the edges of vegetation (Swift 1998; Roche et al. 2014; Dietz and Kiefer 2016; Jones and Froidevaux 2023). In terms of habitat, the Brown long‐eared bat is typically associated with woodlands, including coniferous and broadleaf forests, but the species can also be found in riparian areas, treelines, scrubs, and orchards (Swift 1998; Roche et al. 2014). In contrast, the Soprano pipistrelle is not always closely associated with woodlands, although it can be found in broadleaf woodlands. The species shows a particular preference for riparian habitats and low‐density urban areas (Roche et al. 2014; Jones and Froidevaux 2023).

Metabarcoding and next‐generation sequencing techniques have revolutionised the investigation of diet composition across carnivorous, herbivorous, omnivorous, and insectivorous species (Shehzad et al. 2012; García‐Estrada et al. 2012; Gill et al. 2023; Groen et al. 2023; Schumm et al. 2023; Tosa et al. 2023; Zurdo et al. 2023). These methods enable comprehensive dietary analysis, surpassing traditional studies that rely on morphological prey identification. This approach, now widely applied in bat studies (Galan et al. 2018; Alberdi et al. 2020; Tiede et al. 2020; Tournayre et al. 2021; Curran et al. 2022; Bourlat et al. 2023), provides comprehensive dietary profiles across numerous samples with high accuracy.

To provide a comprehensive understanding of the diets of the Brown long‐eared bat and Soprano pipistrelle across Ireland, we collected faeces from multiple maternity roosts over 3 years at three sampling periods each year: gestation, lactation, and post‐lactation. We used metabarcoding and NovaSeq Illumina sequencing to identify prey and bat DNA from these faeces. Specifically, we (1) examined the full range of prey taxa consumed by the Brown long‐eared bat and Soprano pipistrelle across 12 sampled roosts in Ireland, (2) compared the bat–prey interaction patterns between the Brown long‐eared bat and Soprano pipistrelle and their variation among roosts, and (3) compared the richness and composition of their diets across different roosts, years, and bat reproductive periods to identify the primary factors influencing dietary variation.

## 2 Materials and Methods

### 2.1 Bat Faeces Collection

#### 2.1.1 Identification of Key Bat Roosts

Bat Conservation Ireland provided a list of 33 potential roosts for both the Brown long‐eared bat and Soprano pipistrelle. For each maternity roost, details were recorded, including building type, town, county, Irish grid coordinates, bat species present, feasibility of faecal sample collection (if available), and other relevant information. From this list, maternity roosts were pre‐selected based on the following criteria: ease of access to faeces without disturbing the bats, likelihood of obtaining high‐quality samples, and the characteristics of the surrounding landscape. A total of 19 maternity roosts–15 for Brown long‐eared bats and four for Soprano pipistrelle–were sampled over a 3‐year period (2021–2023).

#### 2.1.2 Bat Faeces Sampling Protocol

Bat faeces were collected once per reproductive period–gestation, lactation, and post‐lactation–over a 3‐year period, from mid‐May to the end of August in 2021, 2022, and 2023, from all pre‐selected roosts. Sampling periods were chosen based on the life cycle of the two bat species (Aughney et al. 2018; Russo 2023). All collections were approved by the Department of Housing, Local Government and Heritage of Ireland under licence numbers DER/BAT 2021‐39, DER/BAT‐2022‐48. Additionally, specific permits were obtained from the National Parks and Wildlife Service (NPWS) permits of Connemara and Wicklow Mountains National Parks. Old droppings were removed from the roost floor. Fresh faecal samples were collected over 2 days from newspaper sheets placed directly on the floor of bat maternity roost attics, using sterilised tweezers (Puechmaille et al. 2007). This non‐invasive sampling method avoided handling bats directly. All collected faeces were stored in individual tubes with silica beads to prevent DNA degradation (Taberlet et al. 1999; Puechmaille et al. 2007), and were then stored at −20°C. Where indoor collection was not feasible, an outdoor collection system was established to gather droppings. Environmental conditions, including weather and wind, were assessed beforehand to optimise sample collection. Samples were collected immediately after sunrise each morning to minimise DNA degradation, again using sterilised tweezers and placed into individual tubes with silica beads.

**FIGURE 1:**
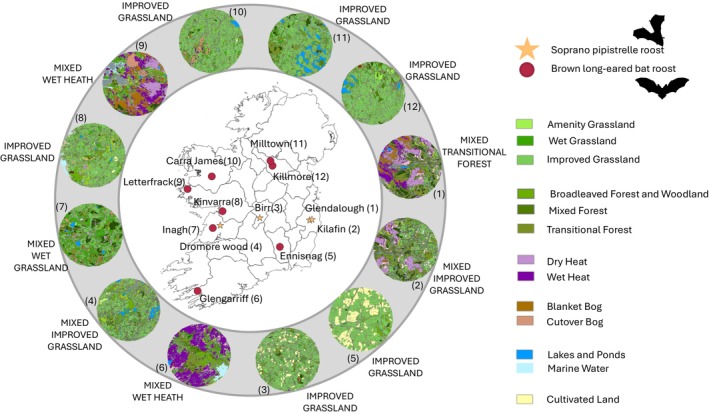
Location and land cover type of the roosts included in the analysis. 5 km buffer for Soprano pipistrelle roosts: Glendalough (Co. Wicklow) (1), Kilafin (Co. Wicklow) (2), Birr (Co. Offaly) (3), Dromore Wood (Co. Clare) (4). 3 km buffer for Brown long‐eared bat roosts: Ennisnag (Co. Kilkenny) (5), Glengarriff (Co. Cork) (6), Inagh (Co. Clare) (7), Kinvarra (Co. Galway) (8), Letterfrack (Co. Galway) (9), Carra James (Co. Mayo) (10), Milltown (Co. Cavan) (11), Killmore (Co. Cavan) (12). Data of land cover was extracted from the National Land Cover Map of Ireland (EPA and Tailte Éireann 2023).

Because non‐target bat species were observed in some of the maternity roosts sampled, a protective measure was implemented to minimise cross‐contamination: only isolated faecal samples were collected. Droppings touching each other were excluded from sampling to ensure the reliability of the DNA analysis. To be included in the study, a roost had to provide faecal samples at each of the three sampling periods annually. Consequently, only eight Brown long‐eared bat roosts met this criterion for 3 years of sampling. For Soprano pipistrelle roosts, two were included in 2021 and sampled for all 3 years, with two additional roosts sampled in 2022 and 2023. Therefore, this study included data from a total of 12 bat maternity roosts (Figure 1). A total of 4627 faecal samples were collected over the 3 years, comprising 3566 faeces from Brown long‐eared bat roosts and 1061 faeces from Soprano pipistrelle roosts (Table S1 presents details of the number of faecal samples collected for each roost).

### 2.2 Land Cover Data

The National Land Cover Map of Ireland (EPA & Tailte Éireann 2023) was used to gather information on the landscape characteristics surrounding each maternity roost (Tailte Éireann Permit No. CYAL50442193). The distance of foraging sites from the roost varies between the two bat species. For the Brown long‐eared bat, according to the literature, the foraging site distances range from a radius of 0–3.3 km from the roost (Entwistle et al. 1996; Swift 1998; Dietz and Kiefer 2016; Aughney et al. 2011; Ashrafi et al. 2013; Hillen and Veith 2013). Therefore, we defined the potential foraging areas to be within a 3 km radius from the roost. In contrast, for the Soprano pipistrelle, the range is broader, varying from 480 m to 12.3 km from the roost (Davidson‐Watts and Jones 2006; Nicholls and Racey 2006; Stone et al. 2015; Dietz and Kiefer 2016). Within Ireland, Bat Conservation Ireland has typically recorded Soprano pipistrelles foraging 5 km from their roost (Tina Aughney pers. com). Therefore, we estimated the foraging area to be within a 5 km radius. Land cover data were extracted using *QGIS Desktop 3.36.0* for buffers of 3 km and 5 km around the respective roosts of the two species (Figure 1).

### 2.3 Preparation of Sequencing Libraries

DNA was extracted and sequenced from a maximum of 20–25 faecal samples per roost and sampling event, when available. Each faecal sample was processed individually, to maintain sample‐level resolution. Full details of the number of faecal samples collected per roost and reproductive period are provided in the Table S1. DNA extraction was conducted annually after each fieldwork season. To avoid any cross‐contamination across roosts, DNA extractions were processed by roost in 96‐well plates. Arthropod and bat DNA were extracted following Zarzoso‐Lacoste's protocol (2018) with modifications (Table S2). To minimise cross‐contamination during the extraction process, all extractions were conducted in a fume hood, with UV sterilisation for 45 min before and after each extraction batch, and all reusable material was washed with a 10% bleach solution. Two primer sets targeting mitochondrial DNA Cytochrome *c* oxidase subunit I (COI) (Folmer et al. 1994; Hebert et al. 2003) were used to identify bat and prey species from extracted DNA. These primer sets, fwhF1/fwhR1 (Vamos et al. 2017) and ZBJ‐ArtF1c/ZBJ‐ArtR2c (Zeale et al. 2011) amplify partial COI regions of 178 and 157 bp, respectively. Tagged PCR was performed (Bohmann et al. 2022) with forward and reverse primers tagged with 8 bp unique sequences to allow for multiplexing of the faecal samples, following Taberlet et al. (2018) and Illumina recommendations. Each 96‐well plate included one negative extraction control, one negative PCR control, and one positive PCR control. To minimise contamination, PCRs were conducted in a different laminar fume hood to that used for the DNA extractions, which was also UV‐sterilised for 45 min before and after the PCR plate. For each PCR amplicon, the concentration was estimated using the QUBIT hight‐sensitivity DNA protocol. PCR amplicons were pooled in equimolar concentrations into sequencing libraries by roost and year of sampling, generating a total of 74 libraries over the 3 years of the study. Pooled libraries were cleaned up using the AMPure XP Beads protocol (Beekman coulter).

The libraries were sent to Biomarker Technologies for Illumina NovaSeq sequencing on the NovaSeq 6000 platform, using 150 bp paired‐end runs. Illumina adapters and indexes were added using ligation‐based library preparation to minimise tag‐jumping (Bohmann et al. 2022).

### 2.4 Recovery of Bat and Prey Sequences

The identification of bat and prey species from NovaSeq sequences was conducted using the pipeline proposed by Browett et al. (2021) in combination with OBITools v1.2.13 (Boyer et al. 2016), VSEARCH (Rognes et al. 2016), and BLAST v2.12.0 (Camacho et al. 2009) scripts. Additionally, R scripts from the Metabarpark Project (Wangensteen and Turon 2017) were used to adapt file formats between successive steps. Each library was processed separately. Briefly, raw sequences from each library were ‘paired‐ended’ using ‘*illuminapairedend*’ with a minimum score of 40. The next step was ‘*ngsfilter*’ used to demultiplex the sequences based on 8 bp sample indices and attribute them to the corresponding faecal samples. Sequences with unexpected lengths, those containing ‘N’ base, or those occurring less than 10 times were removed (Bernard et al. 2021; Perea et al. 2024). Additionally, chimeric sequences were identified using the ‘*uchimeout’* function. The sequences that remained after filtering, representing Molecular Operational Taxonomic Units (MOTUs), were clustered with 98% identity using the ‘*sumaclust*’ command. MOTUs were assigned to taxonomic identifiers (taxid), using remote BLAST against the National Center for Biotechnology Information (NCBI) database with 10 sequences targeted by MOTUs (Altschul et al. 1990; Sayers et al. 2022), with a 98% identity and 90% coverage threshold (Tournayre et al. 2021). Where MOTUs were assigned to several species, the most recent common ancestor was selected (Wangensteen et al. 2018). The complete script is available in the Supporting Information Bioinformatic Script.

To minimise potential false positives resulting from cross‐contamination, MOTUs present in extraction or PCR blanks were removed from the library if their percentage of reads in blanks exceeded 10% compared to other samples (Wangensteen et al. 2018; Vescera et al. 2024). Additionally, a stringent threshold was applied to eliminate potential artefact sequences introduced during PCR and sequencing. MOTUs with occurrence levels below 1% in each faecal sample were removed (Mata et al. 2021) using the ‘*hill_div*’ function from the *hilldiv* R package (version 1.5.1, Alberdi and Gilbert 2019). The taxonomy of each MOTU was retrieved using the Taxonomizr R package v0.10.6 (Sherrill‐Mix 2023).

The third step involved identifying the sequence belonging either to ‘host’ (bat species), ‘prey’ (species considered as prey), ‘external parasite’ (arthropod species determined as a parasite of the bat species), or ‘other’ (any other categories of species). The presence or absence in Ireland of each identified species taxa was verified using the National Biodiversity Data Center database (National Biodiversity Data Center 2024) and scientific literature (O'Connor et al. 2009; Ashe et al. 2012; O'Connor and Nelson 2012; Baker et al. 2013; Chandler 2024). Only species documented as present in Ireland in at least one of these databases were included for the final analysis. The current known distribution of prey species not recorded in Ireland was verified using the Global Biodiversity Information Facility (GBIF) database (GBIF 2024).

Finally, during sampling, multiple bat species were observed sharing roosts, and eventually, non‐target bat species were observed urinating on newspapers placed to collect faecal samples, potentially introducing their DNA into faecal samples. DNA from urine was considered acceptable for analysis because it did not affect diet analysis results. However, a threshold was set to ensure analysis integrity. Only faecal samples containing at least 90% DNA from a single bat species were included, balancing data reliability with sample retention.

### 2.5 Statistical Analysis

Statistical analyses were conducted using R (version 4.3.3, R Core Team 2022) and R Studio (version 2023.12.1.402). The *ggplot2* package (version 3.5.1; Wickham 2016) was used for data visualisation. Following recommendations by Galan et al. (2018), PCR products with less than 500 reads were discarded. Faecal samples from each roost and sampling event were sequenced, with the number of samples varying by event. For sampling events with more than 20 faecal samples, a random selection of up to 20 samples was made using the ‘*sample*’ function to ensure unbiased representation.

To evaluate the reliability of the data, *‘iNEXT’* and ‘*ggiNEXT*’ functions of the *iNEXT* package (version 3.0.1) were used to generate sample completeness curves for each sampling event (Chao et al. 2014; Hsieh et al. 2016). These curves estimate sample coverage, which quantifies the proportion of the total species diversity in a community that is represented in the sample.

Prey richness in each faecal sample was calculated using the ‘*hill_div*’ function from the *hilldiv* package (version 1.5.1), with *q* set to 0. To compare prey richness between the Brown long‐eared bat and Soprano pipistrelle, we performed a Wilcoxon–Mann–Whitney test (Wilcoxon 1947; Mann and Whitney 1947). Additionally, we used the Kruskal–Wallis test to assess differences in prey richness across roosts, sampling time points, and years (Nwobi and Akanno 2021).

To explore the specificity of foraging patterns among bat maternity roosts, we assessed diet distinctiveness (DD) between roosts as the one minus the pairwise Pianka's niche overlap (Pianka 1973; Roswag et al. 2018; Mata et al. 2021), using the ‘niche.overlap’ function from the *spaa* package (version 0.2.2, Zhang 2016). Pairwise Pianka's niche overlap values were verified using the bootstrap function ‘*niche.overlap.boot*’ from the *spaa* package. To visualise interaction patterns between prey arthropod order and maternity roosts, we then generated web plots using the ‘*bipartiteD3*’ function from the *bipartiteD3* package (version 0.3.2, Terry 2024) for the two bat species.

To investigate the effects of spatial (maternity roost location) and temporal (year and sampling event) factors on prey composition and dispersion, we first created a distance matrix using the ‘*vegdist*’ function with the Jaccard method based on the presence/absence of prey from the *Vegan* package (version 2.6.6, Oksanen et al. 2024). We then performed a permutational multivariate analysis of variance (PERMANOVA) to assess differences in beta diversity (i.e., variation in prey composition) among roosts, years, and events, applying the ‘adonis2’ function with 999 permutations (Anderson 2006, 2014; Anderson et al. 2006). To visualise compositional patterns between bat species and roost, we generated a non‐metric multidimensional scaling (nMDS) plot using the ‘metaMDS’ function (Tong 1992; Zuur et al. 2007). To assess whether roost or year had a stronger influence on dietary composition, we constructed a dendrogram using Jaccard dissimilarity and complete linkage clustering with the ‘*hclust’* function from the *stats* package (v3.6.2). Finally, to examine the relationship between prey composition and landscape diversity, we performed a Mantel test based on Spearman correlation using the ‘*mantel*’ function (Borcard and Legendre 2012; Tournayre et al. 2021).

## 3 Results

### 3.1 Landscape Characteristics

According to the National Land Cover Map of Ireland, both Brown long‐eared bat and Soprano pipistrelle maternity roosts were situated within a mix of natural or semi‐natural habitats. Across all roosts, grassland was the predominant habitat, averaging 47.6% of the surrounding area. Most roosts had improved grassland exceeding 24%, and in six roosts, it accounted for more than 50% of the area within the buffer. Only three roosts, all located in protected areas, had relatively low grassland cover (< 15%) (Figure 1). The percentage of land cover levels 1 and 2 is available in Data Set S1 on DataDryad.

### 3.2 Prey and Bat Species Identification

The 74 NovaSeq sequencing runs generated a total of 476,391,107 reads, with individual runs producing between 3,903,247 and 14,480,797 reads. Raw data and metadata are available on DataDryad (DOI: https://doi.org/10.5061/dryad.qbzkh18vt). After applying bioinformatic filtering, 255,022,827 reads were retained for further analysis. After filtering out MOTUs with less than 1% occurrence across faecal samples, we identified 907 taxa at the species level. Each species was assigned to a category (prey, bat host, external parasites, or other), resulting in a total of 691 prey species identified for both the Brown long‐eared bat and Soprano pipistrelle. Of these, 51 species were not previously recorded in Ireland and were excluded from the final analysis. The overall list of the 907 identified species, along with the 51 non‐Irish species and their known distributions, is provided in Data Set S2 on DataDryad.

In terms of host species, five bat species were identified: Brown long‐eared bat (1257 faeces), Soprano pipistrelle (475 faeces), Lesser horseshoe bat ( *R. hipposideros* ) (168 faeces), Natterer's bat ( *Myotis nattereri* ) (44 faeces), and common pipistrelle (*Pipistrelle pipistrellus*) (14 faeces). The Brown long‐eared bat was identified as the host with 100% confidence for 1163 faeces (93% of the faecal samples). These numbers represent only the samples confidently assigned to a species following quality control; additional collected samples either failed to amplify, produced too few reads, or could not be confidently assigned. For faecal samples containing DNA from multiple bat species, 53% were identified as containing Brown long‐eared DNA at 98% or 99% confidence. Similarly, the Soprano pipistrelle was identified as the host in 460 faeces (97% of the samples assigned to this species) with 100% confidence. Among the faecal samples containing DNA from other bat species, 41% showed Soprano pipistrelle DNA at 98% or 99% confidence.

Sampling coverage was estimated across all sites and years, using the iNEXT and ggiNEXT functions. All roosts exceeded 91%, indicating a high level of data reliability. However, when coverage was assessed at each roost for individual sampling events, variability was observed, with some sampling periods showing lower coverage (Figure S1).

### 3.3 Factors Influencing Prey Richness in the Diet

**FIGURE 2:**
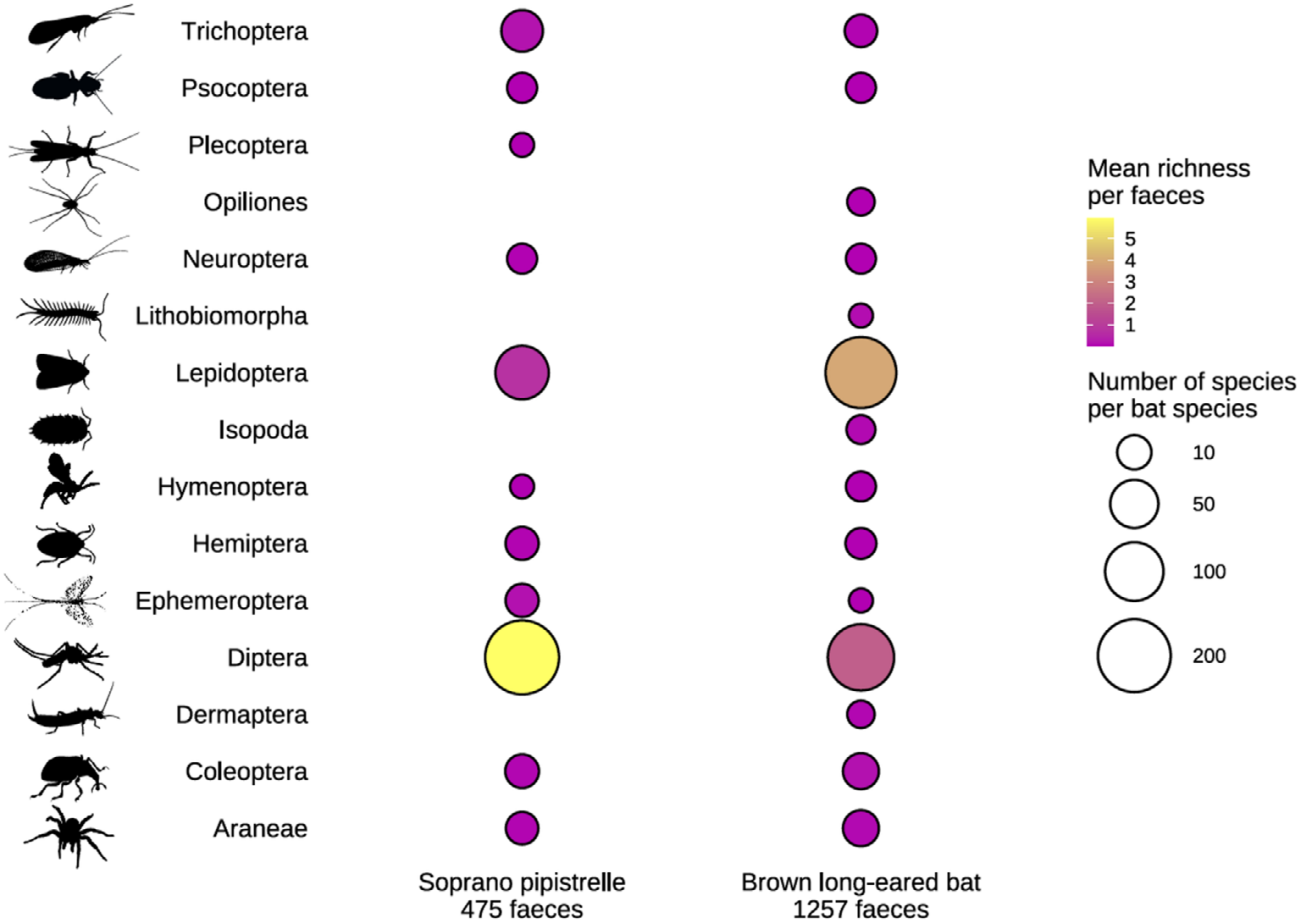
Dot plot showing total prey species by order in the diet of Brown long‐eared bat and Soprano pipistrelle, along with the mean species per order.

**FIGURE 3:**
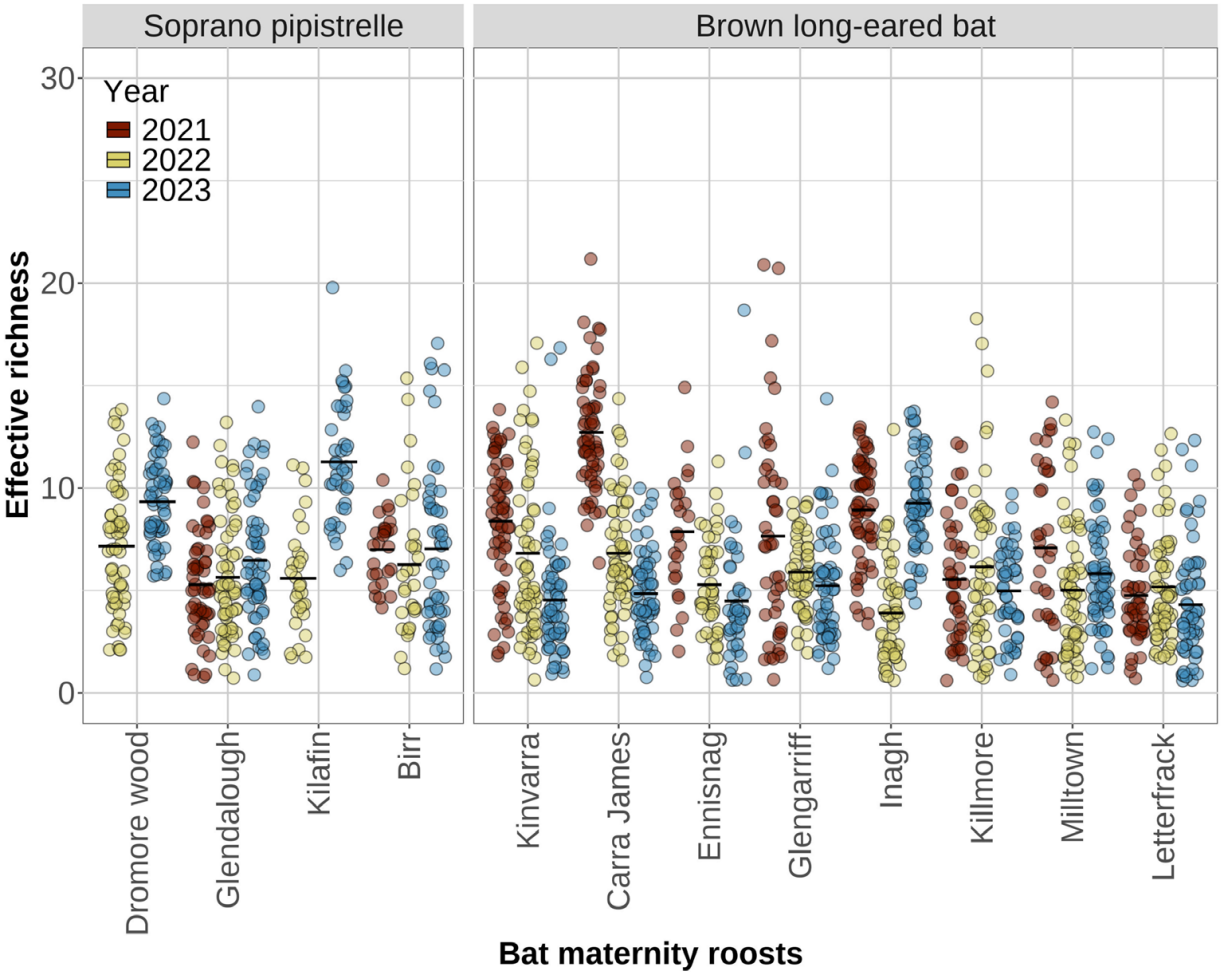
Jitter plot showing the effective richness for each faecal sample analysed per bat species and bat maternity roost. Colours represent the year of sampling collection. Effective richness represents the number of prey species per faecal sample. The black line indicates the mean per year and roost.

A total of 16 orders were identified in the diets of the Brown long‐eared bat and Soprano pipistrelle, with 14 and 11 arthropod orders, respectively. Lepidoptera and Diptera species were the most prevalent prey for the Brown long‐eared bat, with 184 and 154 species, respectively. The diet of the Soprano pipistrelle was largely dominated by Diptera species (210 species), while 83 species of Lepidoptera were recorded (Figure 2). Prey richness per faecal sample ranged from 1 to 21 species for the Brown long‐eared bat and from 1 to 20 for the Soprano pipistrelle, with an average of 6.3 and 7.06 species, respectively (Figure 3). This difference in prey richness between the two species was statistically significant (*p* = 3.483e‐16; Table 1). Within both bat species, prey richness varied significantly across the roosts and from year to year (Figure 3). Although prey richness differed across years, there was no significant difference between sampling periods (Table 1).

**TABLE 1:** Results of comparison Kruskal–Wallis tests for comparing prey species richness for each variable: Bat species, roost, year, and sampling period.

Bat species	Variable tested	Kruskal–Wallis test results		
		*χ* ^2^	df	*p*
Brown long‐eared bat	Year	112.67	2	**< 2.2E‐16**
	Roost	105.73	7	**< 2.2E‐16**
	Sampling period	4.22	2	0.121
Soprano pipistrelle	Year	47.16	2	**5.76E‐11**
	Roost	59.27	3	**8.41E‐13**
	Sampling period	0.52	2	0.771
Brown long‐eared bat and Soprano pipistrelle	Bat species	21.53	1	**3.48E‐06**

*Note:* Significant *p*‐value (*p*‐value < 0.05) is indicated in bold.

The mean richness of arthropod orders identified per faecal sample varied across the sampling period and roost for both bat species. In the diet of the Brown long‐eared bat, richness of the two dominant orders–Lepidoptera and Diptera–showed variation across both roosts and sampling time points, with no single arthropod order consistently dominating. Lepidoptera richness showed strong variation across roosts, with a mean number of species per faecal sample ranging from 0.27 to 12.00 and a maximum richness of 17 species, while the Diptera order presented a mean richness ranging from 0.15 to 6.00. In contrast, in Soprano pipistrelle roosts, Diptera species consistently had the highest prey richness, with mean values per roost ranging from 2.80 to 10.45, and a maximum richness of 17 species. Lepidoptera, the second dominant order, showed lower mean richness, ranging from 0.05 to 4.50, with a maximum of seven in one of the roosts sampled. Other arthropod orders showed low mean richness across all roosts, all below 1.00 (Figure S2).

### 3.4 Bat and Prey Interactions

**FIGURE 4:**
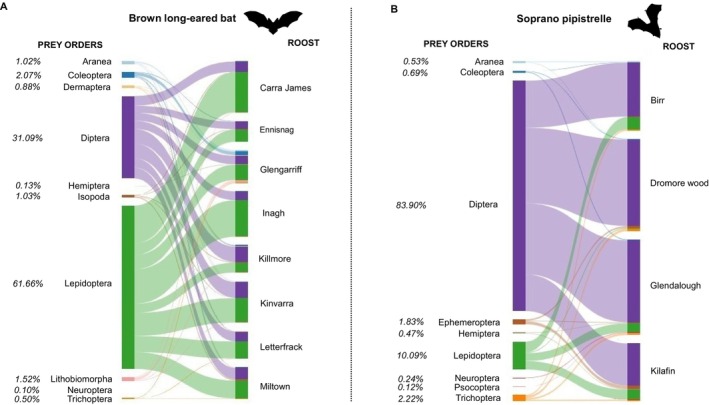
Web plots showing interactions between prey orders with Brown long‐eared bat (A) and Soprano pipistrelle (B) across maternity roosts. Percentages show the percentage of each order in diets. Only orders representing more than 0.1% of the total interactions are presented. The web plot was created using the ‘bipartiteD3’ function from the bipartiteD3 package.

Typically, DD values range from 0 (low dietary distinctiveness) to 1 (high distinctiveness). DD between Brown long‐eared bat and Soprano pipistrelle was 0.82, indicating limited overlap in prey composition. Pianka's niche overlap value was 0.172, with a bootstrap estimate of 0.182, providing confidence in this result. Web plot analysis revealed that the Soprano pipistrelle's diet was primarily dominated by Diptera, which accounted for 83.9% of prey interactions. In contrast, the Brown long‐eared bat's diet was more balanced, with Lepidoptera (61.66%) and Diptera (31.09%) being both dominant prey orders (Figure 4).

At the family level, both bat species interacted with a wide range of Lepidoptera and Diptera families. The Brown long‐eared bat interacted with 21 Lepidoptera and 29 Diptera families, while the Soprano pipistrelle interacted with 21 Lepidoptera and 35 Diptera families. However, most of these families contributed only a small proportion to the overall interactions. Among the Brown long‐eared bat's interactions, the most frequently encountered Lepidoptera families were Noctuidae (68.50%), Hepialidae (9.66%), Geometridae (8.93%), and Erebidae (8.58%), while 17 other families each contributed less than 5%. Within Diptera, Muscidae (13.38%) and Scathophagidae (9.83%) were present, but Tipulidae (57.06%) was dominant, representing the majority of interactions. The remaining 26 Diptera families contributed less than 5% each. In the Soprano pipistrelle's diet, the primary Lepidoptera families were Blastobasidae (28.15%), Hepialidae (16.13%), Noctuidae (13.49%), Tortricidae (12.02%), Geometridae (7.04%), and Gracillariidae (6.45%), while 15 additional Lepidoptera families contributed less than 5% each. Among Diptera, the dominant families were Psychodidae (23.28%), Chironomidae (22.96%), Limoniidae (14.81%), and Tipulidae (7.13%), with 29 other families each representing less than 5%. Detailed information on the contribution of each family to the diet is provided in Figure S3.

Within bat species, DD values between roosts ranged from 0.258 to 0.740 for the Brown long‐eared bat, and from 0.400 to 0.662 for the Soprano pipistrelle. Most interspecific roost comparisons showed high DD values, with the exception of one Soprano pipistrelle roost (Birr), located in an improved grassland landscape. This roost exhibited moderate dietary dissimilarity with several Brown long‐eared bat roosts, with DD values ranging from 0.557 (Ennisnag; improved grassland) to 0.857 (Glengarriff; mixed wet heath and woodland) (Table 2). The Brown long‐eared bat exhibited variability in interactions across roosts, with Lepidoptera and Diptera contributing between 35.10% to 80.02% and 19.29% to 55.18% of the total interactions with prey species, respectively. At the Glengarriff roost, three additional arthropod orders each accounted for more than 5% of the interactions: Araneae (5.10%), Coleoptera (10.51%), and Lithobiomorpha (8.17%). Similarly, the Soprano pipistrelle's interactions with Lepidoptera and Diptera varied across roosts, ranging from 1.01% to 17.63% and 72.86% to 93.64%, respectively. However, Diptera consistently dominated the diet, accounting for more than 70% of prey interactions at all roosts. Ephemeroptera contributed more than 5% of the diet at one roost, Kilafin, representing 5.82% of the total interactions (Figure 4).

**TABLE 2:** Diet distinctiveness values between all roosts included in the studies, Brown long‐eared (in black) and Soprano pipistrelle (in blue).

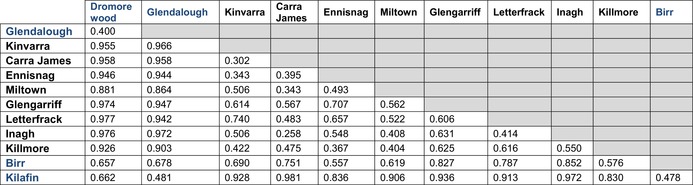

*Note:* Diet distinctiveness between each roost was estimated as minus one the Pairwise Pianka's niche overlap value.

### 3.5 External Factors Influencing Beta Diversity

**FIGURE 5:**
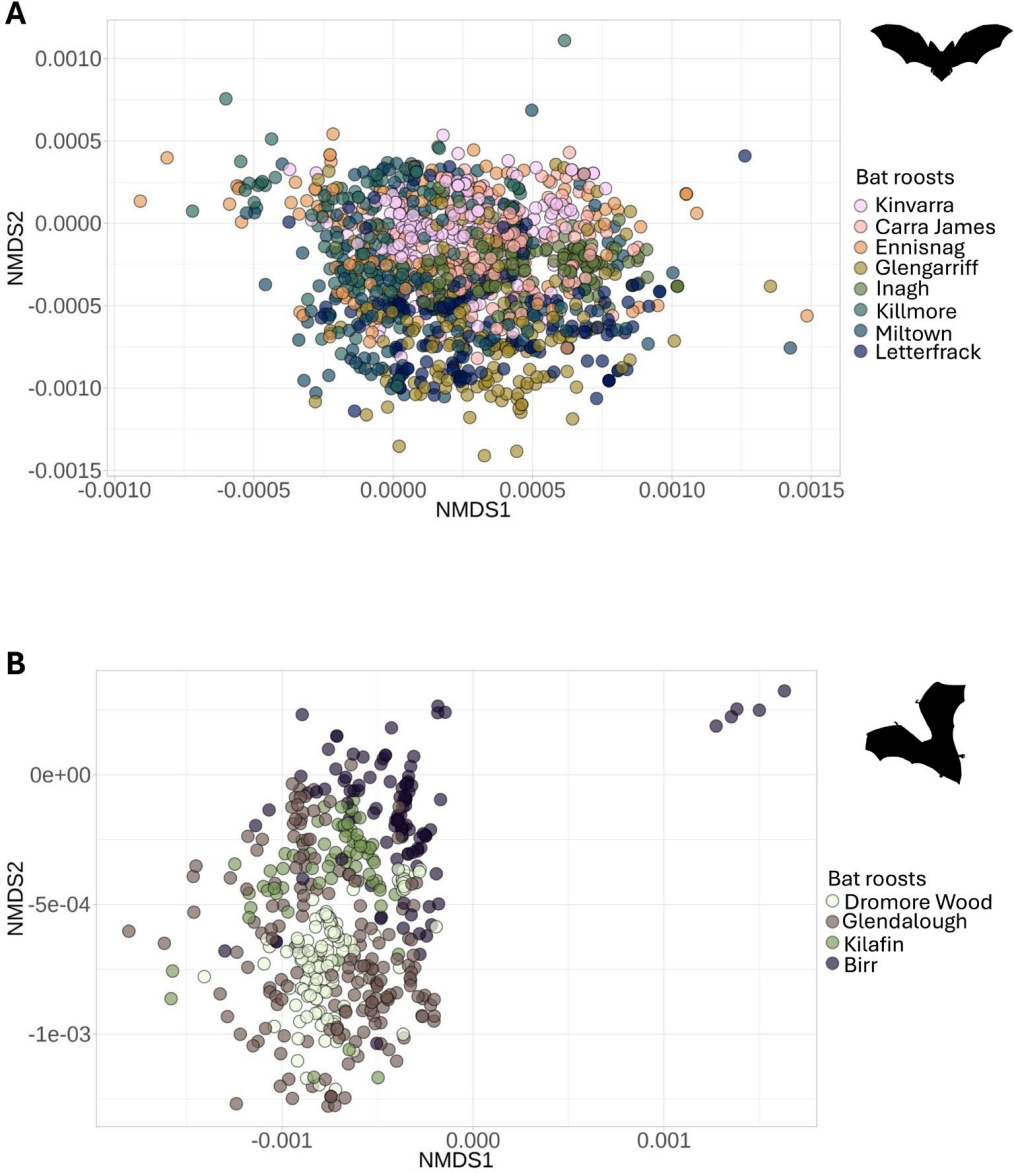
Non‐metric multidimensional scaling plot showing the dissimilarity in diet composition across bat maternity roosts of Brown long‐eared bat (A) and Soprano pipistrelle (B). Each point represents a faecal sample, and the distance between points reflects the similarity of species composition based on Jaccard dissimilarity. In the legend, numbers correspond to roost IDs as shown in Figure 1. The stress value of 0.08 indicates a good representation of the data in two dimensions. Dissimilarity matrix used to create the plot was performed using ‘vegdist’ function of Vegan package. Plot was created with ‘metaMDS’ function. For better readability two samples were taken out the plot in (A), the original plot is available in Supporting Information S4.

Initial nMDS analysis revealed the presence of outliers, which were subsequently removed to ensure a more robust and accurate assessment of beta diversity. A total of 13 outliers were removed. The revised results showed a better fit of the ordination, indicating improved clarity in the separation of prey composition between bat species and roosts. The list of outliers is available in Table S3. Diet composition, based on presence/absence data, significantly varied depending on bat species and spatial and temporal factors. PERMANOVA results revealed that the prey composition varied significantly between bat species, bat roosts, sampling periods, and year (Table 3). The nMDS plot showed clear separation between bat species, but with variability across roosts within bat species. The stress value of 0.08 indicated a good fit for the ordination (Figure 5 and Figure S4).

**TABLE 3:** Results of PERMANOVA (A) and Mantel tests (B), used to identify significant differences in diet composition between bat species, maternity roosts, year of sampling, and sampling period.

(a)	PERMANOVA based on 999 permutations			
	df	*R* ^2^	*F*	Pr(>F)
Bat species	1	0.0563	102.47	**0.001**
Roost	11	0.1866	35.621	**0.001**
Year	1	0.0118	20.582	**0.001**
Sampling period	2	0.0192	16.881	**0.001**
Year × Sampling period	8	0.0486	10.99	**0.001**

(B)	Mantel test based on 999 permutations					
	Statistic *r*	Significance	90%	95%	97.50%	99%
Brown long‐eared bat	0.2525	0.001	0.0107	0.0143	0.0165	**0.0190**
Soprano pipistrelle	0.3161	0.001	0.0060	0.0082	0.0093	**0.0115**

*Note:* Table presents *F*‐statistics, *R* ^2^ values, degree of freedom, and *p*‐values (Pr(>F)). PERMANOVA test (performed using ‘adonis2’ function of Vegan package) compares diet composition between groups. Mantel test measures correlation between beta diversity and landscape diversity, using ‘mantel’ with Spearman correlation. Mantel results are indicated with 95% CI [0.50, 0.75]. Significant *p*‐values (< 0.05) are indicated in bold.

**FIGURE 6:**
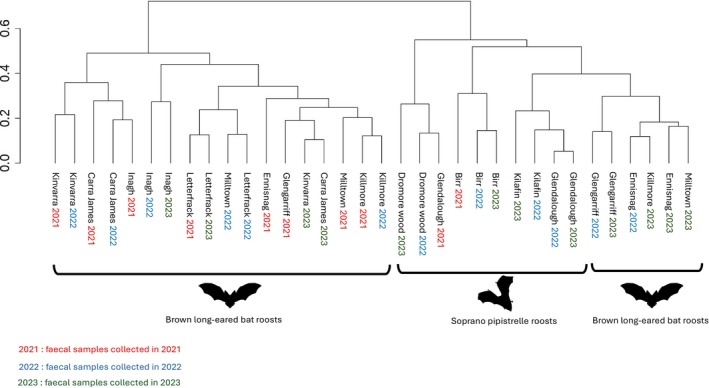
Dendrogram of hierarchical clustering of diet composition across the 12 bat maternity roosts from 2021 to 2023. Clustering was performed using Jaccard dissimilarity and the complete linkage method, with the ‘hclust’ function of the stats package.

Finally, hierarchical clustering of diet composition was performed for the eight Brown long‐eared bat and four Soprano pipistrelle roosts from 2021 to 2023 (Figure 6), using the complete linkage method with Jaccard presence/absence similarity. The analysis showed that the diet compositions of Brown long‐eared bat roosts from 2021 were more similar to each other compared to those from 2022 and 2023. Additionally, the results indicated that diet similarity between years varied depending on the roost. For the Soprano pipistrelle roost in Birr, the clustering suggested greater similarity in diet composition across years compared to other roosts investigated.

Mantel tests revealed a significant positive but low correlation between the dissimilarity of the beta diversity and the dissimilarity of the land cover among roosts for each bat species, with a higher correlation for the Soprano pipistrelle roosts (Table 3). This indicated that the more the landscape surrounding the roost differs, the more the prey composition differs.

## 4 Discussion

The dominance of grassland, coupled with low levels of forest and arable land, makes the Irish landscape distinctive within Europe (Haughey 2021; Eurostat 2024). In our study, the majority of the maternity roosts sampled (i.e., eight Brown long‐eared bat roosts and four Soprano pipistrelle roosts) were located in such grassland‐dominated landscapes, with local variation in the proportion of other semi‐natural habitats. This context provided an opportunity to investigate bat diets in a landscape where grassland dominance shapes ecological interactions.

### 4.1 Brown Long‐Eared Bat's Diet

The Brown long‐eared bat revealed a diverse diet, with 392 taxa identified at the species level across 15 arthropod orders. Lepidoptera was the dominant prey group, comprising 184 species and accounting for 62% of interactions, followed by Diptera with 154 species, which contributed to 31% of interactions. This diversity of arthropods, with both flying and non‐flying prey, such as Aranea, Opiliones, and Lithobiomorpha, is consistent with other metabarcoding studies (Razgour et al. 2011; Andriollo et al. 2019). As Razgour et al. (2011) and Rostovskaya et al. (2000), Noctuidae species were highly represented in the diet, confirming the high ability of the Brown long‐eared bat to successfully prey on eared moth species. Similar to Shiel et al. (1991), this study found that Diptera played an important part in the diet of this species in all roosts. This ability to feed on Diptera species was also confirmed by Roswag et al. (2018). The flexibility in prey selection was further illustrated at one roost location (Killmore), where individuals of the roost fed more on Diptera than Lepidoptera. In highly protected areas like Glengarriff (the roost sampled is located within the Glengarriff Nature Reserve), the diet included a higher proportion of Coleoptera (10.5%), Aranea (5.1%), and Lithobiomorpha (8.2%), indicating that habitat quality might influence prey composition. This flexibility was also reflected in the DD at the bat species level across roosts. Our findings indicate that the Brown long‐eared bat demonstrates flexibility in its diet and suggest that it might adjust it depending on prey availability, as has been proposed by Rostovskaya et al. (2000). Although often considered a Lepidoptera specialist (Dietz and Kiefer 2016; Wilson and Mittermeier 2019; Ancillotto and Russo 2023), our findings support a broader foraging strategy, positioning the Brown long‐eared bat as a generalist with a preference for Lepidoptera.

Prey richness varied significantly between faecal samples, averaging 6.9 species per sample. This is notably lower than the richness observed by Andriollo et al. (2019), who reported between 21.8 and 26.9 species per sample in Switzerland. However, Shiel et al. (1998), found lower prey diversity in *N. leisleri* faecal samples in Ireland compared to those from Germany. These differences in prey richness may reflect unique aspects of the Irish landscape and habitat composition. Nonetheless, order‐level richness was similar between our study and Andriollo et al. (2019) (14 vs. 15 arthropod orders, respectively). This suggests that the main difference may reside in the number of species available within orders, rather than in the diversity of prey types consumed.

### 4.2 Soprano Pipistrelle's Diet

The Soprano pipistrelle exhibited a similarly broad diet, feeding on prey from 11 arthropod orders, with 350 taxa identified at the species level. In contrast to the Brown long‐eared bat, Soprano pipistrelles showed a strong preference for Diptera species, accounting for 60% of the species identified. Food web analyses further emphasised this reliance, as Diptera interactions comprised over 83% of total prey interactions. The prevalent Diptera families included Psychodidae, Chironomidae, Limoniidae, Tipulidae, and Anisopodidae. This aligns with previous findings of Puig‐Montserrat et al. (2020) who also reported a high occurrence of these families in the Soprano pipistrelle diet in Catalogna, Spain. However, unlike this study, which identified Drosophilidae as a dominant component of the diet, our study revealed infrequent interactions with this family in Irish Soprano pipistrelles, detecting it at only two sites. The diet of the Soprano pipistrelle showed notable flexibility, including a wide range of Diptera families and species, suggesting that this bat species may also adjust its diet depending on prey availability. Additionally, while the majority of the prey had small forewing lengths, such as Psychodidae (2–6 mm) and Chironomidae (1–10 mm), larger prey like Tipulidae were also consumed, including species like *Tipula maxima* (22–30 mm), *Tipula paludosa* (13–23 mm), and *Tipula oleracea* (18–28 mm) (Brock 2020). This wide range of prey sizes demonstrates that the Soprano pipistrelle is not limited to a narrow prey size range. Similar patterns have been observed in other small‐bodied bats. For example, the Little Brown Bat ( *Myotis lucifugus* ; head–body length 32–53 mm, comparable to Soprano pipistrelle 36–51 mm; Wilson and Mittermeier 2019) is known to forage on species of Cicadidae (*Magicicada cassini*) up to 28 mm in body length (Isenhour et al. 2024). Such cases suggest that predator body size alone is not the primary factor limiting prey size in insectivorous bats (IIsenhour et al. 2024). Instead, foraging behaviour, flight, temporal overlap in activity, or prey vulnerability may play a more important role (Denzinger and Schnitzler 2013; Jacobs and Bastian 2016; Puig‐Montserrat et al. 2020). Considering these factors enriches our understanding of bat–prey dynamics by highlighting that ecological context and behavioural flexibility, rather than morphology alone.

Similar to the Brown long‐eared bat, the mean prey richness per faecal sample was relatively low (seven species), though variation was observed across roosts and sampling years. This variation may reflect fluctuations in local prey availability, seasonal shifts in prey populations, or environmental factors influencing foraging behaviour (O'Rourke et al. 2022; Aihartza et al. 2023; Perea et al. 2025)

### 4.3 Influence of Land Cover, Year, and Time

The richness of the bat species' diet was influenced by the species itself, the roost location, and the year of sampling. However, our analyses did not clearly identify which of these factors had the strongest influence on dietary richness, likely due to the limited number of roosts sampled. The variations observed in richness over time might reflect fluctuating prey availability (Clare et al. 2011; Clare et al. 2014; Woodcock et al. 2021) though no clear temporal pattern emerged for either Lepidoptera or Diptera species. A notable observation was made at the Killmore roost, where individuals consumed more Diptera species than at other roosts. This may be attributed to a higher local abundance of Diptera or a lower availability of Lepidoptera, causing a dietary shift. While the land cover around Killmore is dominated by improved grassland (62.5%), similar trends were not observed at other roosts with comparable land cover percentages. Interestingly, bats at this roost consumed more Muscidae and Scathophagidae species, suggesting a roost‐specific influence on diet composition. Improved grasslands are known to support lower arthropod diversity (Arnott et al. 2022), which may contribute to this shift; however, the specific factors behind the increased Diptera consumption at Killmore remain unclear. Importantly, across all sampled roosts and years, the prey composition detected in faecal samples was lower in prey richness compared to studies conducted in more structurally diverse or heterogeneous landscapes (Andriollo et al. 2019; Mata et al. 2021; Tournayre et al. 2021). This suggests that bats foraging in pastureland‐dominated habitats may encounter reduced prey diversity, potentially constraining their foraging behaviour. However, Ireland's comparatively low arthropod diversity relative to other European regions (McCarthy 1986; Nelson et al. 2011; Kelly‐Quinn and Regan 2012; Harrison 2014; Feeley et al. 2020) may also have contributed to these findings. These findings emphasised the importance of considering landscape structure and arthropod biodiversity when assessing bat dietary ecology and support the idea that habitat complexity can enhance prey availability and trophic‐niche width. These results also suggest that prey biomass, rather than the number of prey species, may be the main driver of bat foraging activity.

Roost location also significantly impacted diet composition for both bat species. However, unlike the findings of Tournayre et al. (2021), who reported a strong correlation between landscape dissimilarity and diet composition in greater horseshoe bats ( *Rhinolophus ferrumequinum* ), our results showed only a small positive correlation between these factors. The difference in findings may be attributed to differences in the species studied and their foraging habits.

Hierarchical clustering analysis indicated that the temporal factor (year of sampling) had a notable effect on diet composition. Nonetheless, the relative influence of land cover and year differed among maternity roosts. In some cases, interannual variations had a stronger influence on diet composition than spatial location, while in others, spatial factors were more influential. This suggests that both temporal and spatial factors contribute to shaping bat diets, likely due to their influence on prey availability (Uhler et al. 2021; Saha et al. 2023). A strong impact of temporal dynamics has previously been reported for the Brown long‐eared bat (Razgour et al. 2011; Andriollo et al. 2019). However, our results indicate that at several maternity roosts, the effect of year on diet composition appeared weaker than land cover, suggesting that local habitat characteristics may have a stronger influence on diet patterns than intra‐annual variations in specific contexts.

The key drivers, however, remain unclear and may reflect complex interactions between local prey availability, habitat structure, and bat foraging behaviour. Although our dataset did not directly assess arthropod emergence, the observed variability across roosts, reproductive period and years suggests that both species forage opportunistically, adjusting their diet to the most available prey at a given time and place. This study tends to confirm recent works that have shown that bats adjust their foraging behaviour, including emergence timing and habitat use, in response to prey availability (O'Rourke et al. 2022; Aihartza et al. 2023; Perea et al. 2025). Such dietary flexibility may provide important fitness benefits, particularly for maternity colonies where energetic demands are high (Kunz and Fenton 2005) or when pregnant females or lactating females need to adapt their foraging flight behaviour to carry more weight (Haarsma et al. 2023). From an evolutionary perspective, such plasticity may be under positive selection if it enhances reproductive success under fluctuating resource conditions.

### 4.4 Limitations

Several limitations of this study should be considered, which can be broadly categorised into methodological, database‐related, and ecological constraints.

First, methodological factors may influence our results. Primer choice affects the diversity and composition of prey detected in metabarcoding studies (Alberdi et al. 2018; Tournayre et al. 2020; Browett et al. 2021). We used two widely applied primer sets in bat diet research (fwhF1/fwhR1 and ZBJ‐ArtF1c/ZBJ‐ArtR2c), which provide broad prey coverage and comparability with other bat diet studies but may underrepresent certain arthropod groups (Jusino et al. 2019; Alberdi et al. 2018; Tournayre et al. 2020). However, despite known taxonomic biases, their performance and widespread use support the robustness of our findings. We also used a presence/absence approach rather than relative read abundance. While presence/absence gives more weight to low‐frequency prey taxa (Taberlet et al. 2018; Deagle et al. 2019), read abundance can be biased by DNA extraction, PCR amplification, and sequencing (Elbrecht and Leese 2015; Pawluczyk et al. 2015; Taberlet et al. 2018; Yang et al. 2021; Martoni et al. 2022). Therefore, the presence/absence approach was considered more appropriate. Additionally, stringent bioinformatic filtering excluded MOTUs representing less than 1% of sequences per sample to minimise contamination or secondary predation. While this may have removed some rare prey items (Mata et al. 2021; Alberdi et al. 2018), it likely provided a reliable picture of the main dietary components.

Second, data‐related constraints may affect interpretation. A key limitation lies in taxonomic assignment, which depends on the completeness of reference databases. Although we used two COI gene regions to improve taxonomic resolution, geographic scale can induce intraspecific genetic variation in COI sequences (Bergsten et al. 2012), which may have resulted in some MOTUs being unassigned or misassigned to closely related species. This highlights the need for an Ireland‐specific arthropod DNA database, as current global databases may not fully represent the local biodiversity. Additionally, only species documented as present in Ireland were included in the final analysis. As a result, undocumented prey species may have been excluded from the analysis. Furthermore, our dataset was unbalanced in terms of both the number of roosts sampled per species and the number of faecal samples per roost. While the analytical approach accounts for these differences, some residual effects of sampling imbalance may remain. Finally, sample coverage exceeded 91% overall, but some roosts and sampling events fell below 90%. Lower coverage could have led to a slight underestimation of dietary diversity at certain sites or times.

Third, ecological and other factors should also be acknowledged. First, the absence of prey availability data restricts our ability to directly relate observed dietary patterns to resource use in the surrounding environment. Second, the use of a circular buffer around roosts, while practical for consistent landscape quantification, may not accurately capture the true foraging areas used by individuals. Finally, because all samples were collected from maternity roosts, our dataset likely reflects the diet of adult females and, later in the season, juveniles, with adult males probably underrepresented.

## 5 Conclusion

Using metabarcoding and next‐generation sequencing on faecal samples collected over 3 years with a non‐invasive sampling protocol, this study offers new insights into the diet of the Brown long‐eared bat ( *P. auritus* ) and the Soprano pipistrelle ( *P. pygmaeus* ) in pastureland‐dominated landscapes. We demonstrated that land cover and year significantly influenced prey richness and beta diversity, while the reproductive period affected only beta diversity. While both species demonstrate diet flexibility across roosts, and thus across associated land cover, this flexibility is not expressed to the same degree across arthropod orders. Our results confirm the broad dietary plasticity of the Brown long‐eared bat, whereas the Soprano pipistrelle shows a strong reliance on Diptera, making it potentially more vulnerable in landscapes with reduced prey diversity or abundance. We also found that overall dietary richness was lower than reported in European studies from more structurally complex landscapes.

These findings have important implications in a rapidly changing world, where agricultural intensification, land‐use change, habitat degradation, pesticide use, and climate change are altering arthropod populations and phenology. Understanding bat ecology under such environmental stressors, particularly the degree of foraging plasticity that different species can exhibit, is therefore crucial.

Continued research is essential to enhance our understanding of bat–prey interactions and develop effective conservation strategies. Future studies should combine dietary assessments with direct measures of arthropod abundance and diversity. The development of a comprehensive Irish arthropod DNA database is also essential to improve metabarcoding accuracy. Finally, because bats provide key ecosystem services by feeding on pest species, clarifying the links between bats and these pests across various landscapes could highlight their ecological and economic importance, thereby strengthening the development of conservation strategies.

## Author Contributions

**G. Hurpy:** conceptualization (lead), data curation (lead), formal analysis (equal), funding acquisition (equal), investigation (lead), methodology (lead), project administration (equal), resources (equal), software (lead), visualization (lead), writing – original draft (lead), writing – review and editing (equal). **T. Aughney:** methodology (supporting), resources (equal), writing – original draft (supporting), writing – review and editing (equal). **I. Skujina:** methodology (supporting), project administration (supporting), writing – original draft (supporting), writing – review and editing (equal). **N. Roche:** project administration (supporting), resources (supporting), writing – original draft (supporting), writing – review and editing (equal). **E. C. Teeling:** conceptualization (lead), formal analysis (equal), funding acquisition (lead), methodology (equal), project administration (equal), resources (equal), supervision (lead), visualization (equal), writing – original draft (supporting), writing – review and editing (equal).

## Conflicts of Interest

The authors declare no conflicts of interest.

## Supporting information


**Appendix S1:** Supporting Information.

## Data Availability

Raw sequence and metadata are available on DataDryad (DOI: https://doi.org/10.5061/dryad.qbzkh18vt).
