# Supplementary material for: Metabarcoding Reveals the Dietary Patterns of Bats Within a Unique European Habitat, the Pasture‐Dominated Landscapes of Ireland
Source: Ecol Evol. 2025 Oct 28;15(10):e72310. doi: 10.1002/ece3.72310 (PMC12559813; doi:10.1002/ece3.72310)

Metabarcoding reveals the effect of land cover and temporal variations on the diet of Brown long-eared and Soprano pipistrelle bats within a unique European habitat, the pasture dominated landscapes of Ireland.

Hurpy[1]., G., Aughney[2]. T., Skujina[1,3]. I., Roche[4]. N. and Teeling[1]. E. C*.

[1] School of Biology and Environmental Science, University College Dublin, Belfield, Dublin 4, Ireland.

[2] Bat Eco Services Limited, Virginia, Co. Cavan, Ireland.

[3] Genome Stability Laboratory, School of Biological and Chemical Sciences, University of Galway, University Road, Galway, Ireland.

[4] Bat Conservation Ireland Carmichael House 4-7, North Brunswick Street, Dublin 7, Ireland .

*Corresponding author: emma.teeling@ucd.ie

Table of Contents

Page 2	***Supporting Table S1***
Page 3	***Supporting Table S2***
Page 4	***Supporting Figure S1***
Page 15	***Supporting Figure S2***
Page 16	***Supporting Figure S3***
Page 19	***Supporting Table S3***
Page 20	***Supporting Figure S4***

Supporting Table S1: Number of faecal samples collected at each bat maternity roost over the three years of sampling (2021-2023). *1st sampling period : Gestation ; 2nd sampling period : Lactation ; 3rd sampling period : Post-lactation. Numbers of faecal samples included in the analyses, after that bat species was confirmed, are indicated between parentheses. The sub-total lines show the total of number of faecal samples collected per bat species. The total line presents the combined total for the two bat species. The numbers indicated in the "roost column" correspond to the numbers assigned to each roost in Fig. 1.*

		**2021 sampling collections**			**2022 sampling collections**			**2023 sampling collections**			**TOTAL**
**Bat species**	**Roost**	**1st**	**2nd**	**3rd**	**1st**	**2nd**	**3rd**	**1st**	**2nd**	**3rd**	
**Soprano pipistrelle *Pipistrellus pygmaeus***	Glendalough (1)	16(16)	135(20)	77(20)	25(20)	42(20)	91(20)	23(20)	45(20)	36(20)	**490(176)**
	Kilafin (2)	/	/	/	7(7)	13(13)	11(10)	5(1)	20(19)	23(20)	**79(70)**
	Birr (3)	30(0)	41(12)	36(15)	11(7)	26(13)	15(10)	45(20)	23(20)	12(12)	**239(109)**
	Dromore wood (4)	/	/	/	54(20)	72(20)	29(20)	30(20)	40(20)	28(20)	**253(120)**
	**Sub-total**	**46(16)**	**176(32)**	**113(35)**	**97(54)**	**153(66)**	**146(61)**	**103(61)**	**128(79)**	**99(72)**	**1061(475)**
**Brown long-eared bat *Plecotus auritus***	Ennisnag (5)	31(17)	10(2)	6(5)	43(16)	36(20)	20(6)	17(14)	33(12)	27(15)	**223(107)**
	Glengarriff (6)	39(18)	47(16)	52(12)	19(18)	11(11)	22(20)	50(20)	38(20)	63(18)	**341(153)**
	Inagh (7)	197 (20)	103(20)	97(20)	11(11)	119(20)	55(20)	56(20)	46(20)	48(20)	**732(171)**
	Kinvarra (8)	78(20)	108(20)	168(20)	68(20)	171(20)	76(20)	79(20)	33(20)	71(20)	**852(180)**
	Letterfrack (9)	11(10)	19(18)	182(19)	20(20)	52(20)	64(20)	54(20)	97(20)	45(20)	**544(167)**
	Carra James (10)	43(20)	46(20)	30(20)	26(20)	30(20)	30(20)	33(20)	27(20)	23(20)	**288(180)**
	Milltown (11)	35(19)	26(0)	48(17)	26(20)	30(20)	30(20)	30(20)	34(20)	38(20)	**297(156)**
	Killmore (12)	11(11)	96(16)	17(17)	21(17)	54(20)	22(14)	12(12)	24(16)	32(20)	**289(143)**
	**Sub-total**	**445 (135)**	**455 (112)**	**600 (130)**	**234 (142)**	**503 (151)**	**319 (140)**	**331 (146)**	**332 (148)**	**347 (153)**	**3566 (1257)**
	**TOTAL**	**491 (151)**	**631 (145)**	**713 (165)**	**331 (196)**	**656 (217)**	**465 (201)**	**434 (207)**	**460 (228)**	**446 (225)**	**4627 (1732)**

Supporting Table S2: NucleoSpin Plant kit extraction protocol (adapted from Zarzoso-Lacoste et al., 2018).

**NucleoSpin Plant kit extraction protocol (adapted from Zarzoso-Lacoste *et al.*, 2018)**
**1. Sample preparation**
Add carefully 0.01 gr of pooled faeces into 2mL tube
**2. Cell lysing Buffer PL2 and PL3**
Add 400 µL Buffer PL2 and 10 µL RNase to each tube strips containing faeces sample and close using new cap strips. Mix by vigorous shaking for 15–30 s. Spin for 30 s at 11 000 rpm to collect any sample from the Cap Strips.
Incubate samples at 60°C overnight, on dry bath.
Carefully add 100 µL Buffer PL3 to each sample and close the tubes, mix thoroughly, and incubate for 15 min on ice to precipitate sodium dodecyl sulfate completely.
**3. Filtrate lysate**
Place NucleoSpin filter (violet ring) into new collection tube 2mL
Load all lysate and centrifuge for 2min at 11 000 g.
Collect filtered lysate into a new 1.5 Eppendorf tube and discard filter
**4. Adjust DNA binding condition**
Predispose 450 µL Buffer PC in 1.5mL Eppendorf
Add 400 µL clear lysate and mix thoroughly by pipetting five times
**5. Bind DNA**
Label green column and place it on new column
Transfer 700 µL of lysate to column and centrifuge for 2 min at 14 000 rpm and discard flow through
**6. Wash and dry silica membrane**
Add 400 µL PW1 Buffer and centrifuge for 1 min at 14 000 rpm
Add 700 µL PW2 Buffer and centrifuge for 1 min at 14 000 rpm
Add 700 µL PW2 Buffer and centrifuge for 1 min at 14 000 rpm
Centrifuge at 14 000 rpm for 10 min
**7. Elute DNA**
Place column in labelled 1.5 Eppendorf
Dispose PE Buffer 75 µL into column (pre-heated at 70°C) and incubate at 70°C for 2 min
Centrifuge 2 min at 14 000 rpm
Dispose PE Buffer 75 µL into column (pre-heated at 70°C) and incubate at 70°C for 2 min
Centrifuge 2 min at 14 000 rpm
**Dispose in - 20°C freezer of use for PCR immediately**

Supporting Figure S1. Sample completeness curves for each maternity roost by year and for each sampling period. *Curves were created with iNEXT and ggiNEXT packages, with q = 0. Rarefaction curve (solid line) represents the expected sample completeness as the sample size increases. Extrapolation curve (dashed line) predicts the potential sample completeness beyond the observed sample size. A - H : Brown long-eared bat roosts; I - L : Soprano pipistrelle roosts..*

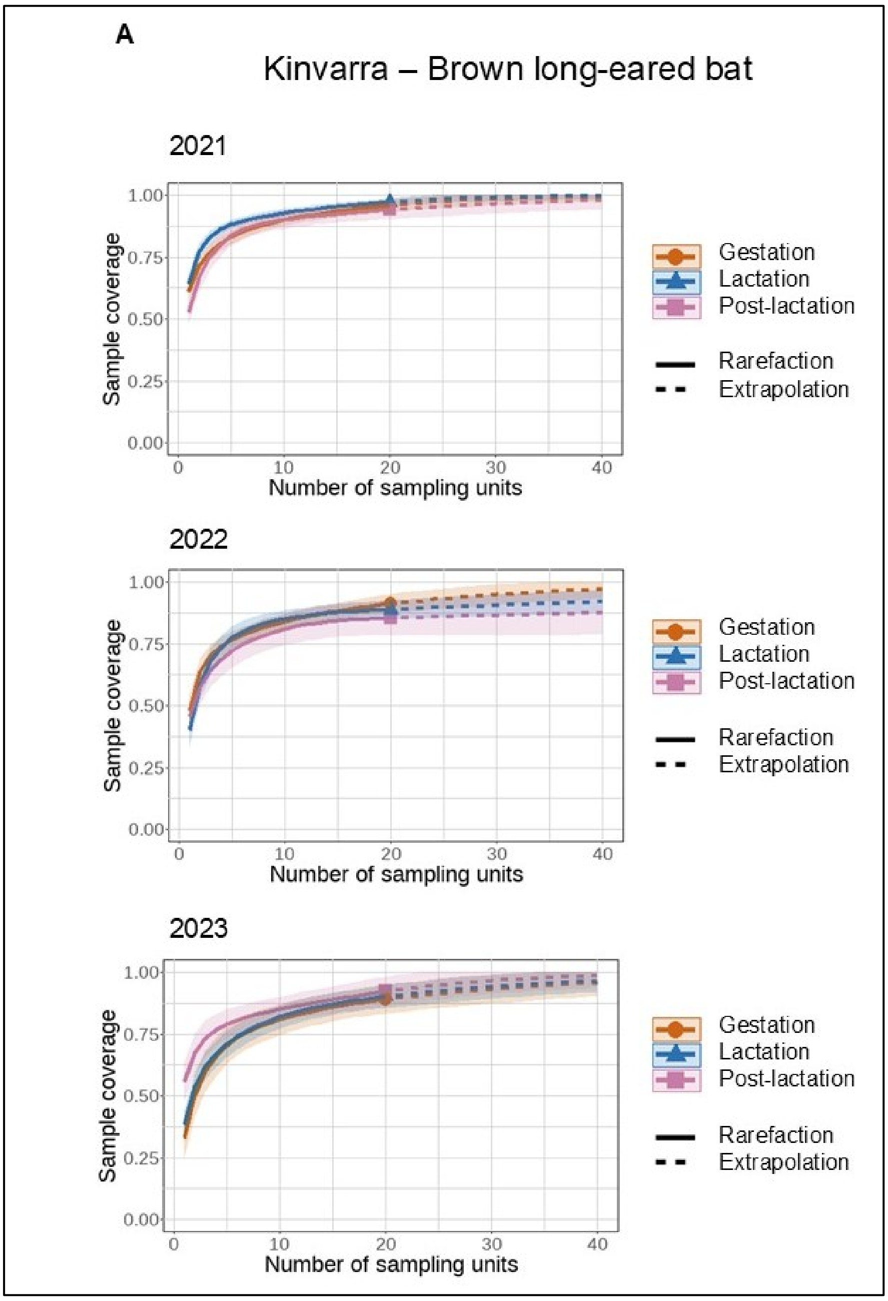

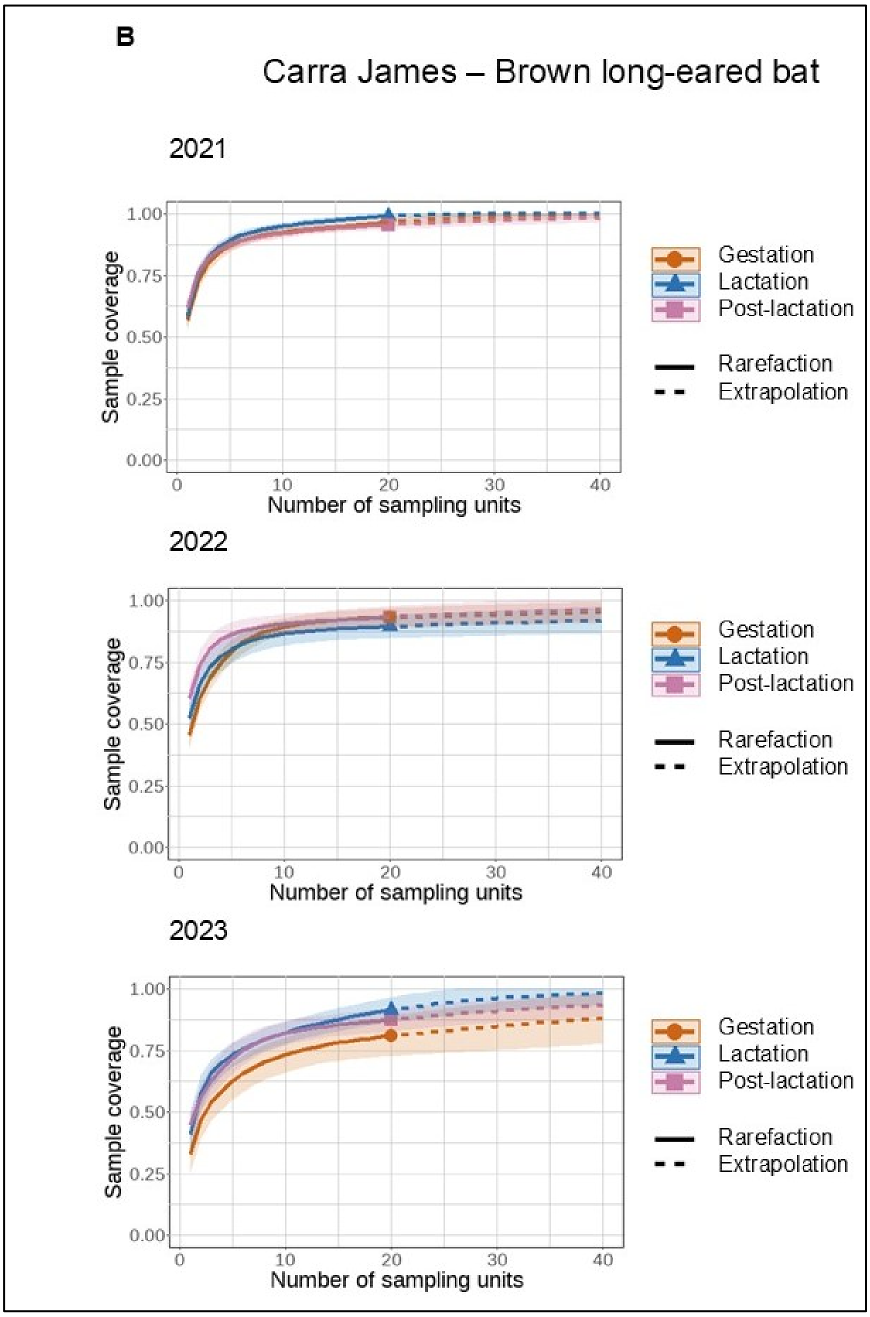
B
Carra James – Brown long-eared bat
2021
2022
2023
Sample coverage
Number of sampling units
0.00
0.25
0.50
0.75
1.00
0
10
20
30
40
Gestation
Lactation
Post-lactation
Rarefaction
Extrapolation

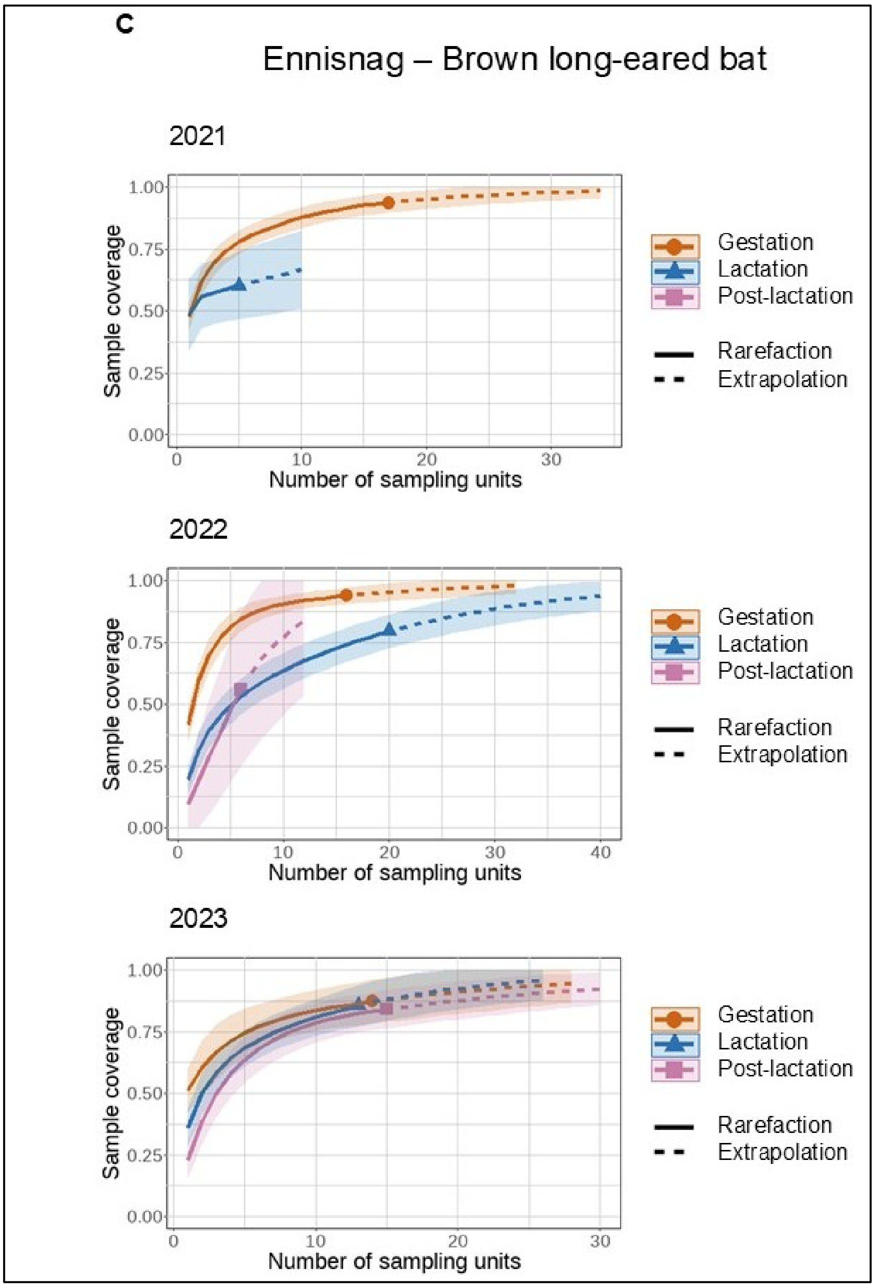
C
Ennisnag – Brown long-eared bat
2021
Sample coverage
1.00
0.75
0.50
0.25
0.00
0
10
20
30
Number of sampling units
Gestation
Lactation
Post-lactation
Rarefaction
Extrapolation
2022
Sample coverage
1.00
0.75
0.50
0.25
0.00
0
10
20
30
40
Number of sampling units
Gestation
Lactation
Post-lactation
Rarefaction
Extrapolation
2023
Sample coverage
1.00
0.75
0.50
0.25
0.00
0
10
20
30
Number of sampling units
Gestation
Lactation
Post-lactation
Rarefaction
Extrapolation

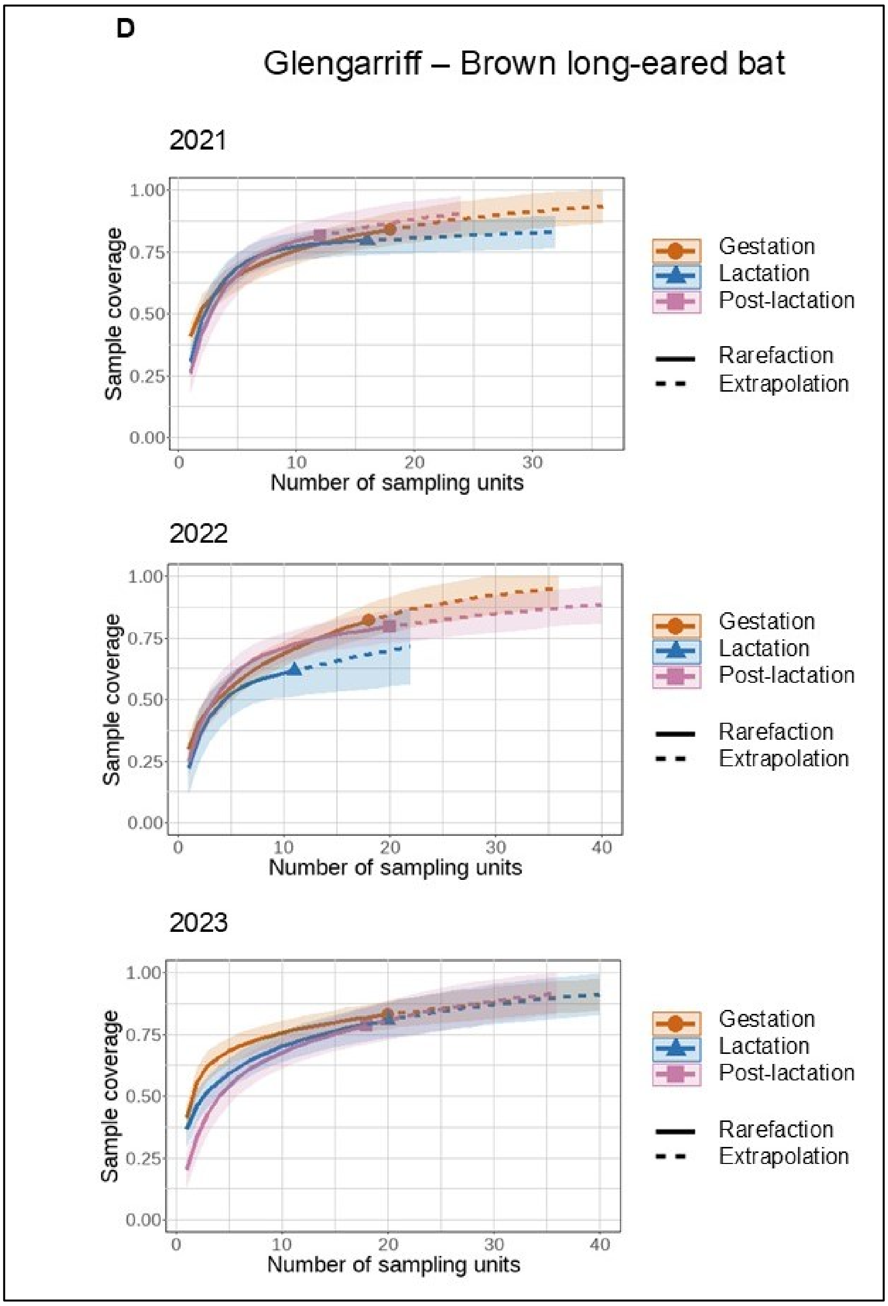
D
Glengarriff – Brown long-eared bat
2021
Sample coverage
1.00
0.75
0.50
0.25
0.00
0
10
20
30
Number of sampling units
Gestation
Lactation
Post-lactation
Rarefaction
Extrapolation
2022
Sample coverage
1.00
0.75
0.50
0.25
0.00
0
10
20
30
40
Number of sampling units
Gestation
Lactation
Post-lactation
Rarefaction
Extrapolation
2023
Sample coverage
1.00
0.75
0.50
0.25
0.00
0
10
20
30
40
Number of sampling units
Gestation
Lactation
Post-lactation
Rarefaction
Extrapolation

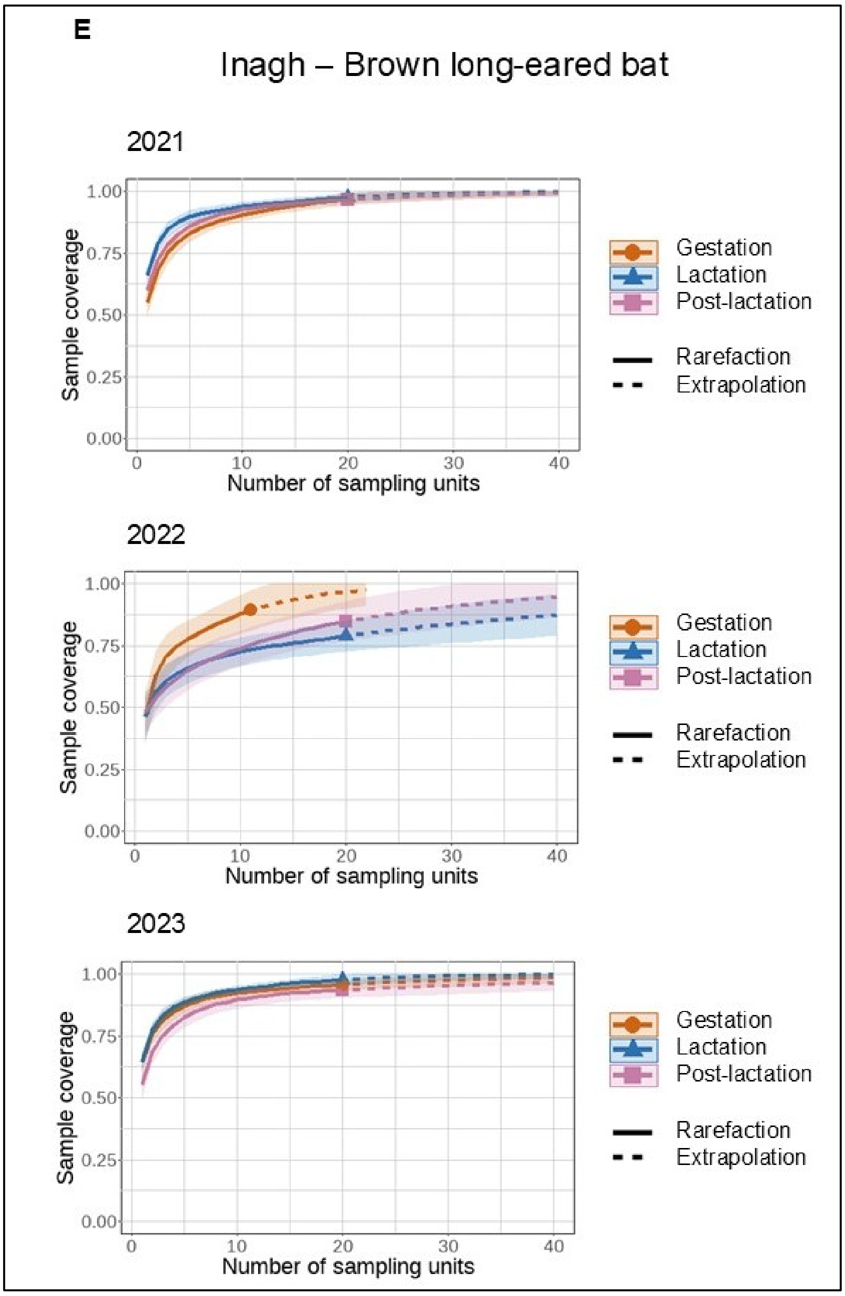
E
Inagh – Brown long-eared bat
2021
Sample coverage
Number of sampling units
Gestation
Lactation
Post-lactation
Rarefaction
Extrapolation
2022
Sample coverage
Number of sampling units
Gestation
Lactation
Post-lactation
Rarefaction
Extrapolation
2023
Sample coverage
Number of sampling units
Gestation
Lactation
Post-lactation
Rarefaction
Extrapolation

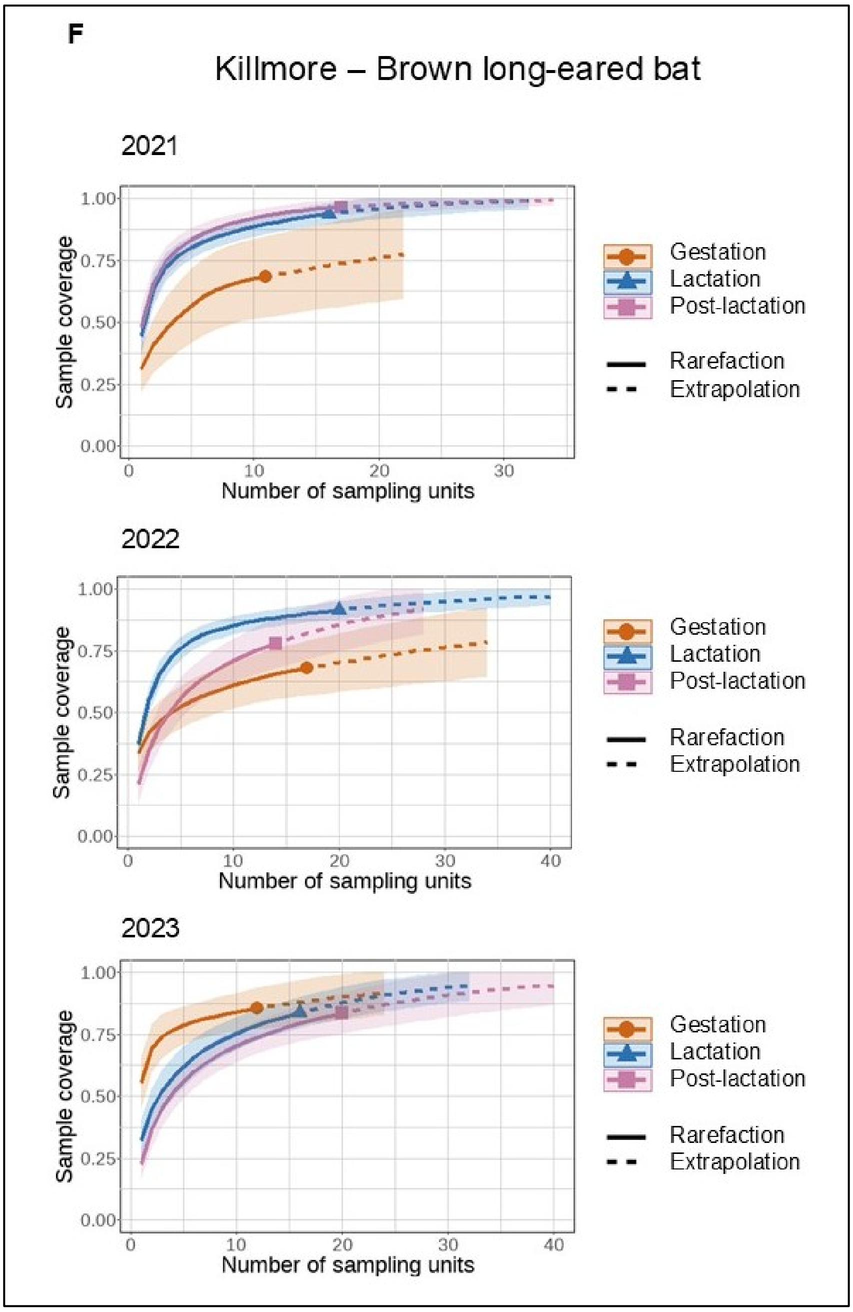
F
Killmore – Brown long-eared bat
2021
2022
2023
Sample coverage
Number of sampling units
1.00
0.75
0.50
0.25
0.00
0
10
20
30
40
Gestation
Lactation
Post-lactation
Rarefaction
Extrapolation

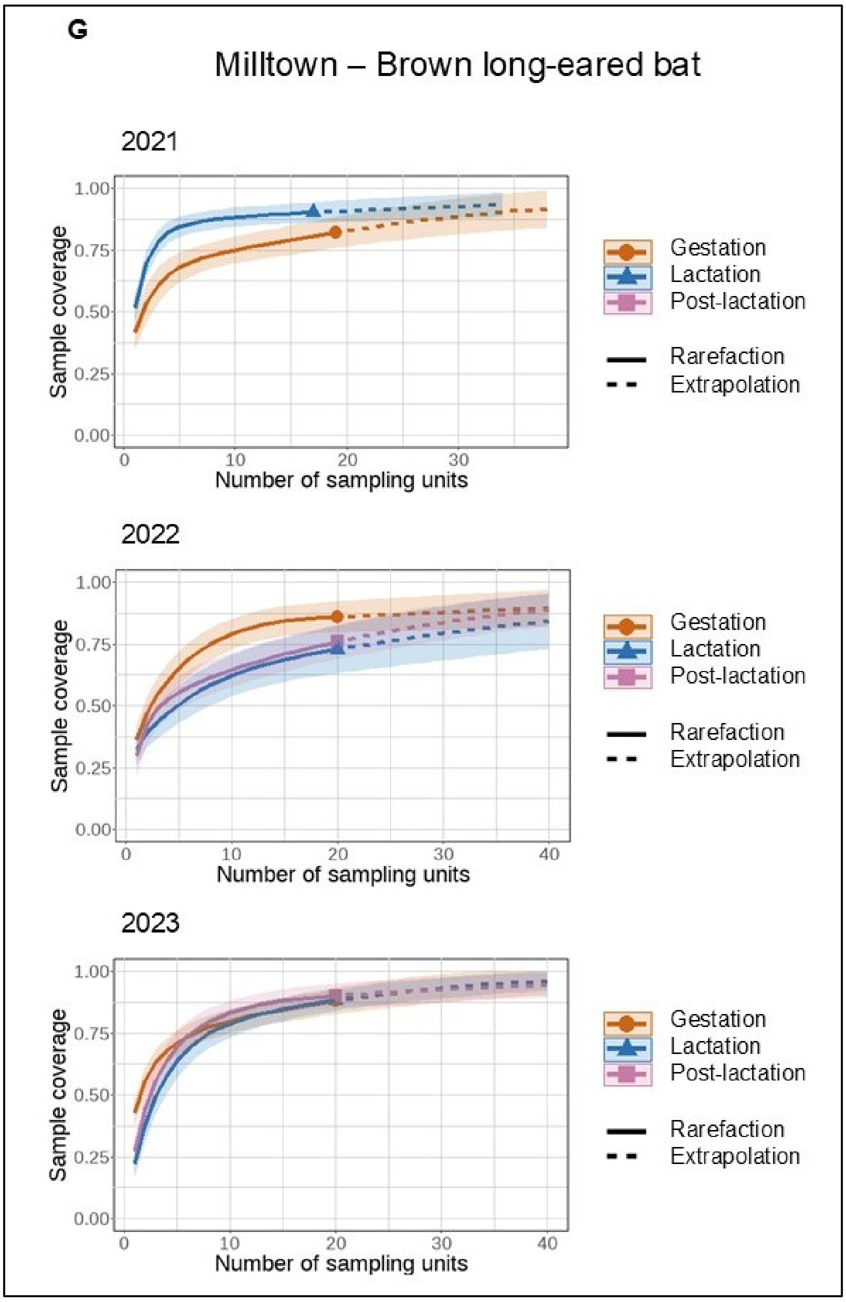
G
Milltown – Brown long-eared bat
2021
2022
2023
Sample coverage
1.00
0.75
0.50
0.25
0.00
0
10
20
30
40
Number of sampling units
Gestation
Lactation
Post-lactation
Rarefaction
Extrapolation

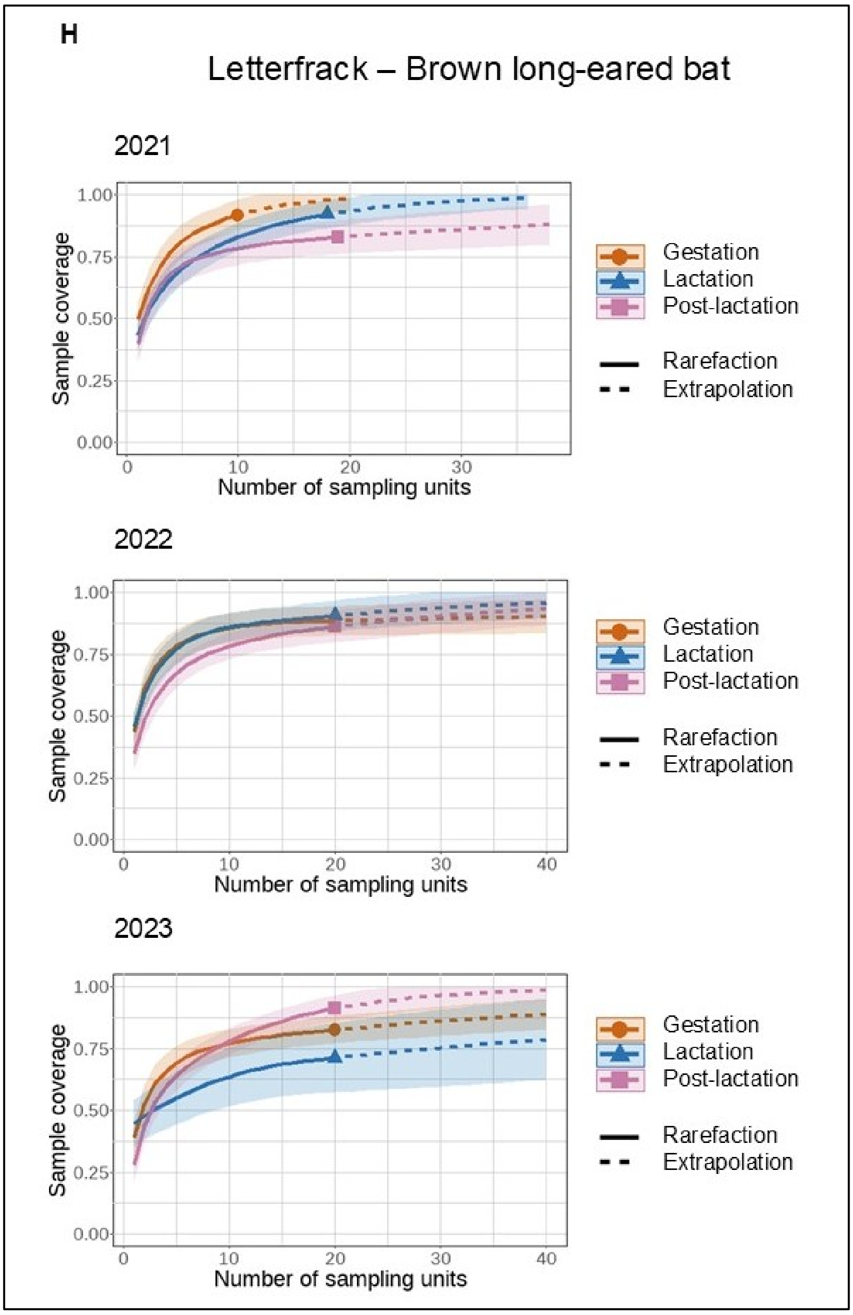
H
Letterfrack – Brown long-eared bat
2021
Sample coverage
1.00
0.75
0.50
0.25
0.00
0
10
20
30
Number of sampling units
Gestation
Lactation
Post-lactation
Rarefaction
Extrapolation
2022
Sample coverage
1.00
0.75
0.50
0.25
0.00
0
10
20
30
40
Number of sampling units
Gestation
Lactation
Post-lactation
Rarefaction
Extrapolation
2023
Sample coverage
1.00
0.75
0.50
0.25
0.00
0
10
20
30
40
Number of sampling units
Gestation
Lactation
Post-lactation
Rarefaction
Extrapolation

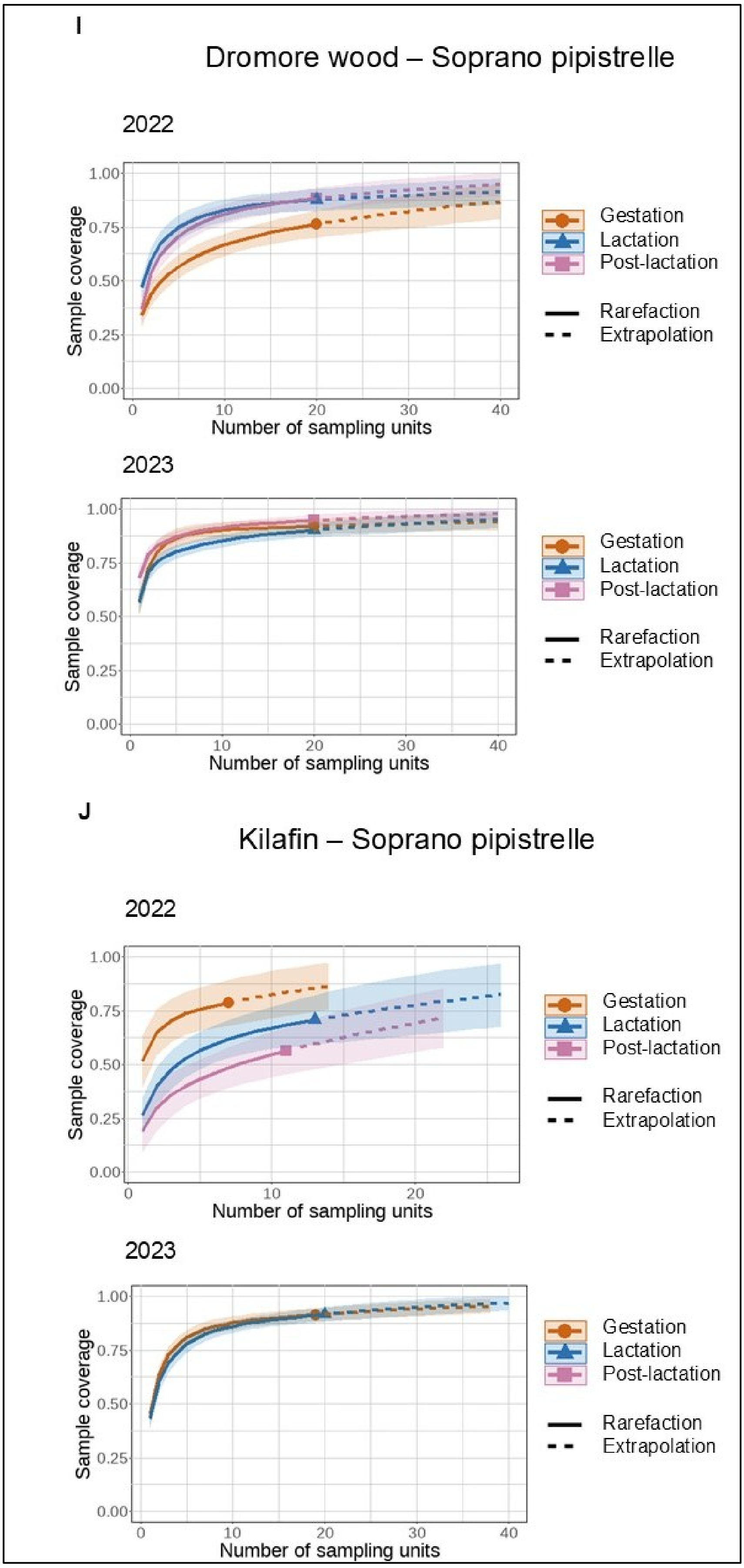
I
Dromore wood – Soprano pipistrelle
2022
Sample coverage
0.00
0.25
0.50
0.75
1.00
0
10
20
30
40
Number of sampling units
Gestation
Lactation
Post-lactation
Rarefaction
Extrapolation
2023
Sample coverage
Number of sampling units
Gestation
Lactation
Post-lactation
Rarefaction
Extrapolation
J
Kilafin – Soprano pipistrelle
2022
Sample coverage
Number of sampling units
Gestation
Lactation
Post-lactation
Rarefaction
Extrapolation
2023
Sample coverage
Number of sampling units
Gestation
Lactation
Post-lactation
Rarefaction
Extrapolation

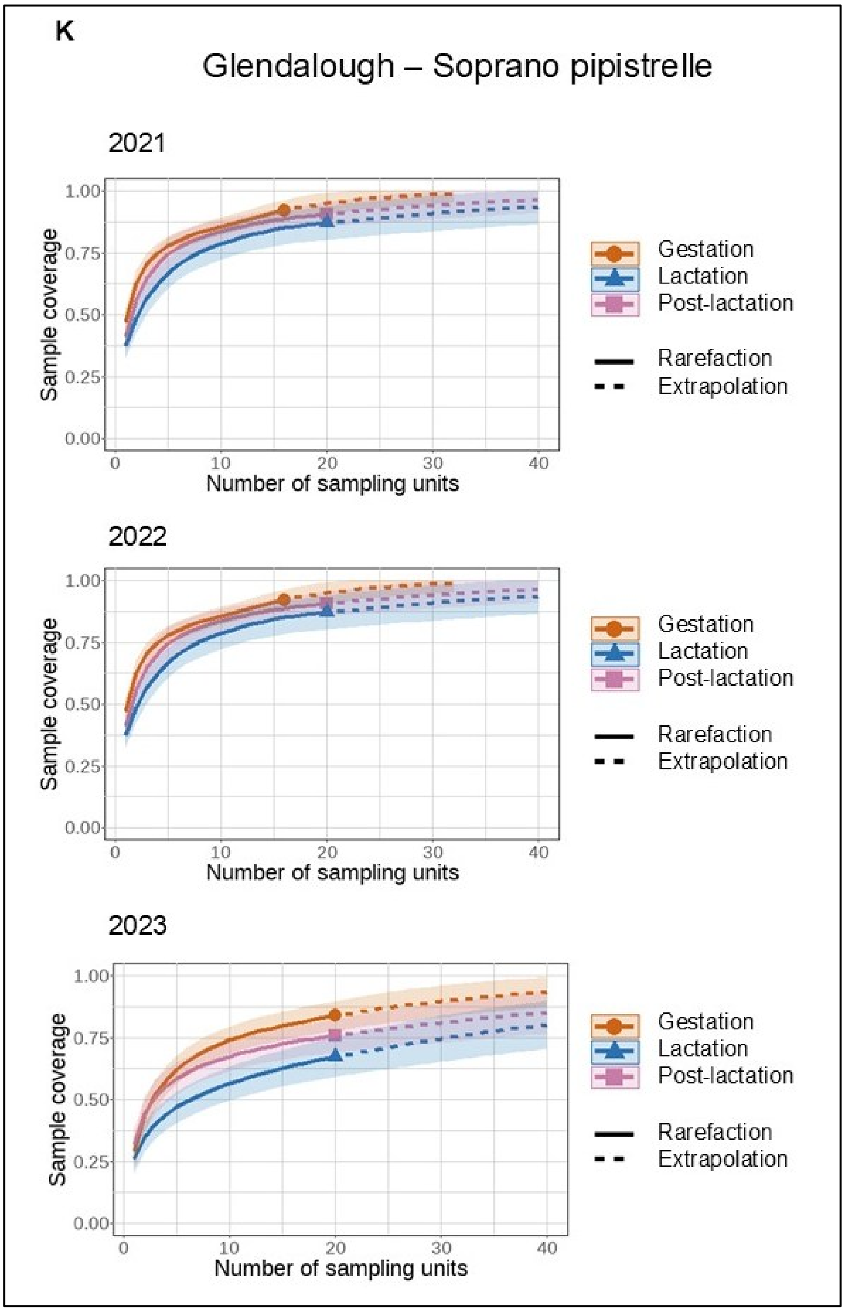
K
Glendalough – Soprano pipistrelle
2021
2022
2023
Sample coverage
Number of sampling units
1.00
0.75
0.50
0.25
0.00
0
10
20
30
40
Gestation
Lactation
Post-lactation
Rarefaction
Extrapolation

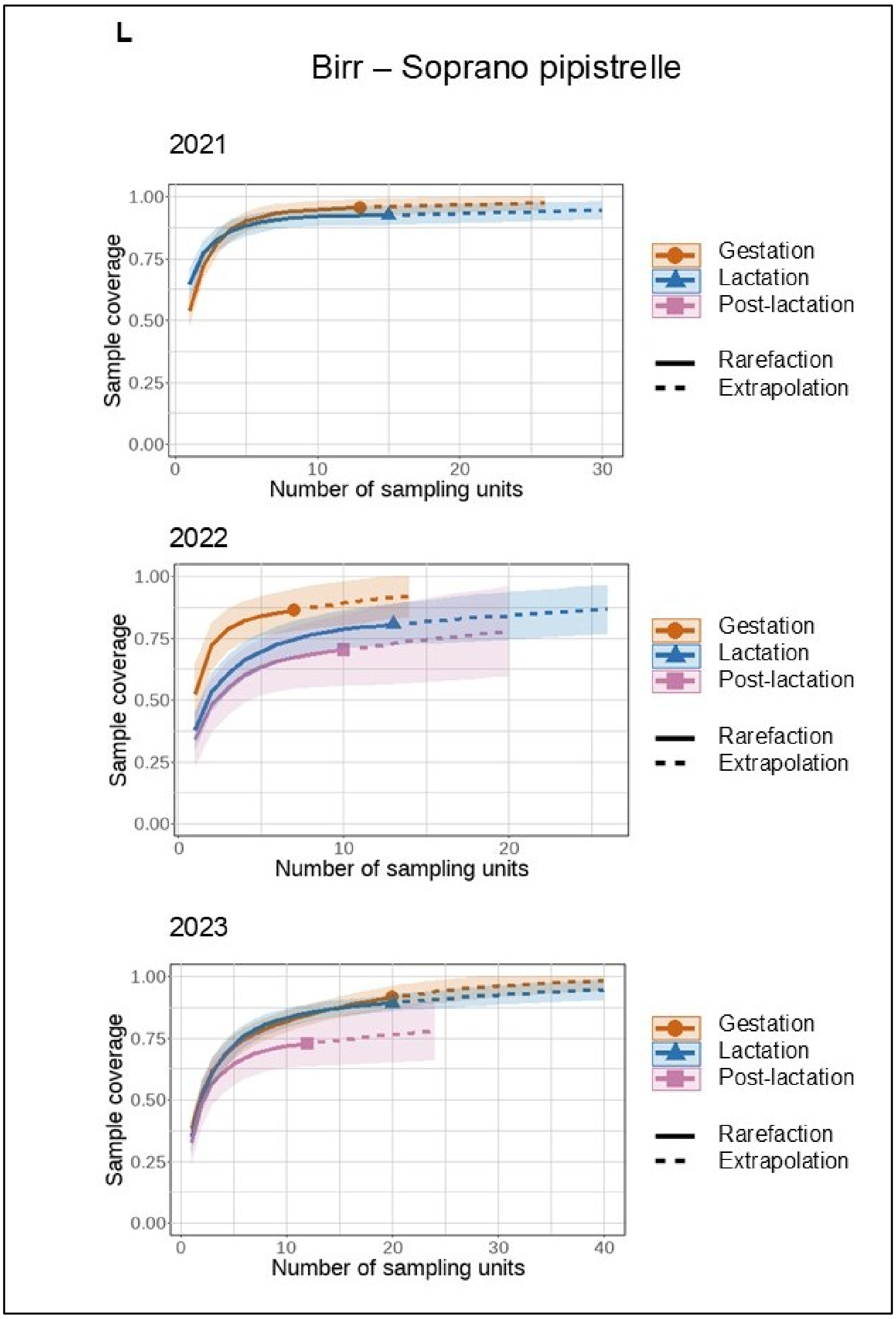
L
Birr – Soprano pipistrelle
2021
Sample coverage
Number of sampling units
Gestation
Lactation
Post-lactation
Rarefaction
Extrapolation
2022
Sample coverage
Number of sampling units
Gestation
Lactation
Post-lactation
Rarefaction
Extrapolation
2023
Sample coverage
Number of sampling units
Gestation
Lactation
Post-lactation
Rarefaction
Extrapolation

Supporting Figure S2

Mean richness of main arthropod orders (Lepidoptera and Diptera) in the diet of Brown long-eared bat (A) and Soprano pipistrelle (B) across all sampling periods*. Horizontal lines at each point represent standard deviation values*

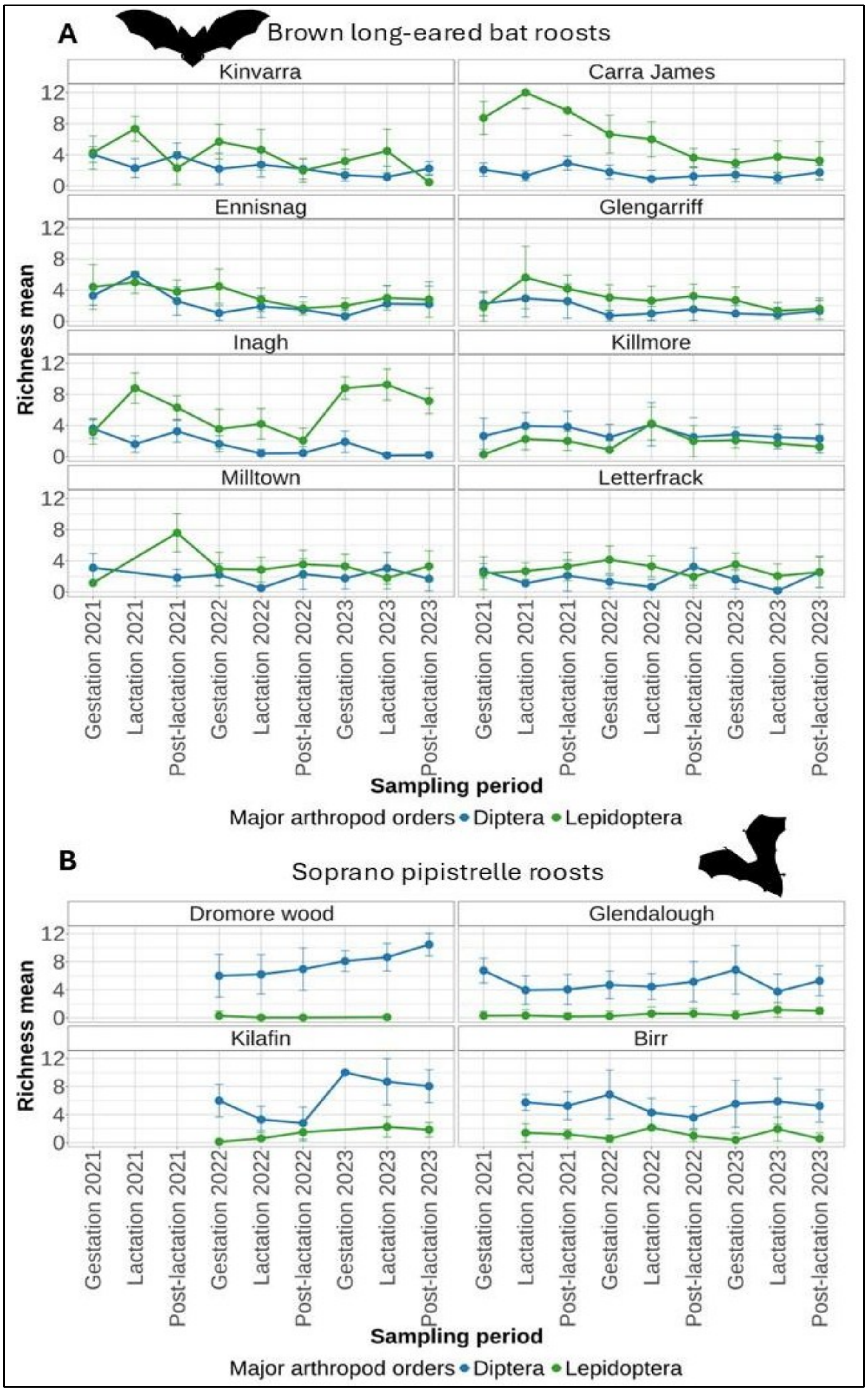

Supplementary Figure S3

Web plots showing interactions between the Brown long-eared bat (S.3A) and Soprano Pipistrelle (S.3B) and prey of Lepidoptera (a) and Diptera (b) families across maternity roosts. *Percentages show the percentage of each order in diets. The web plot was created using the 'bipartiteD3' function from the bipartiteD3 package*.

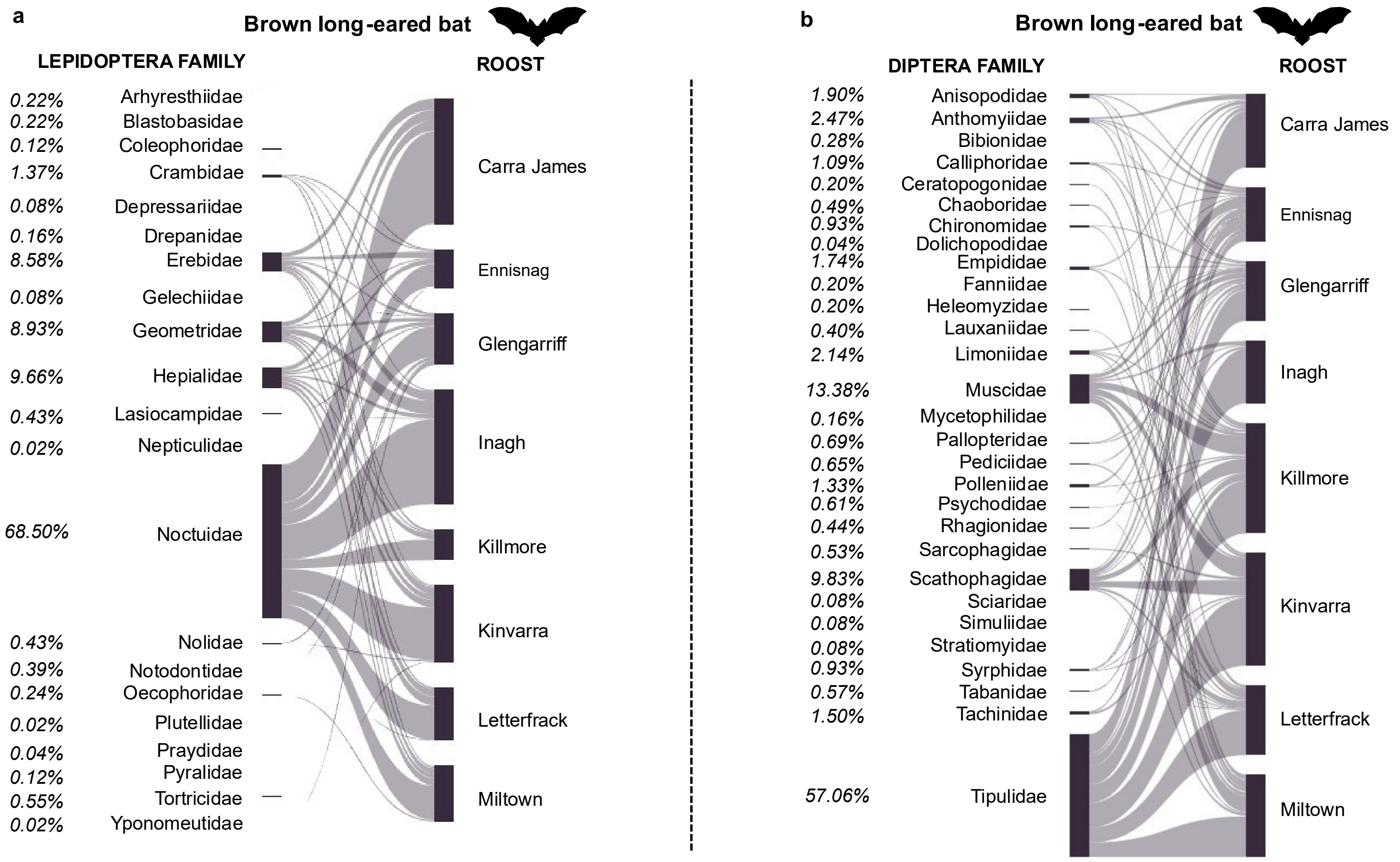

Supplementary Figure S.3A

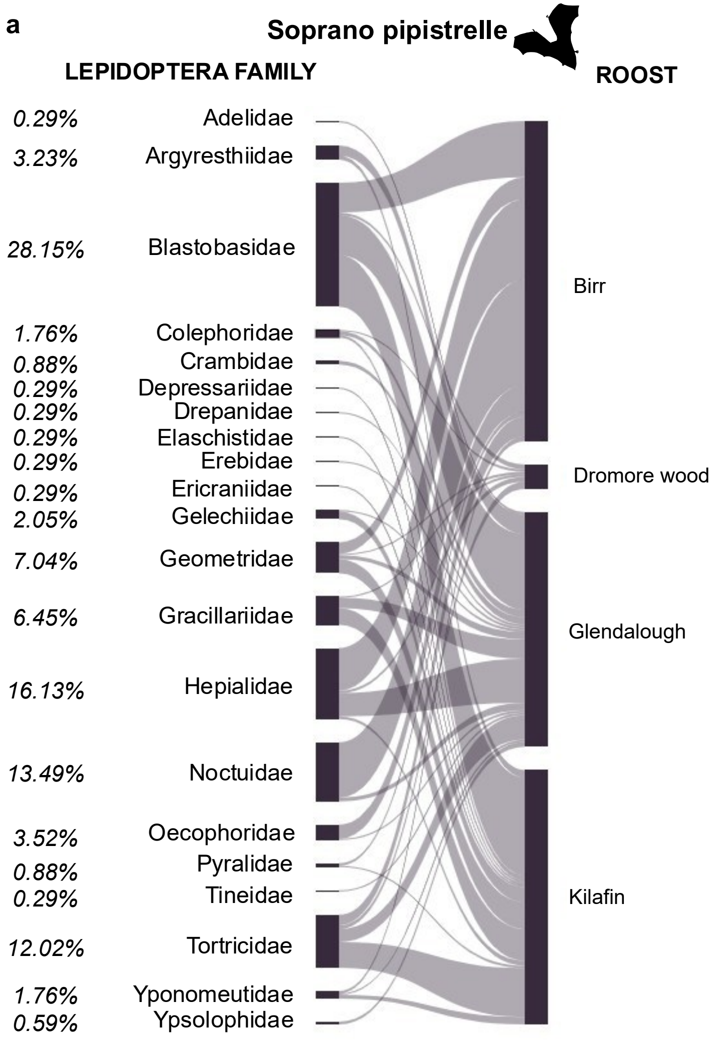

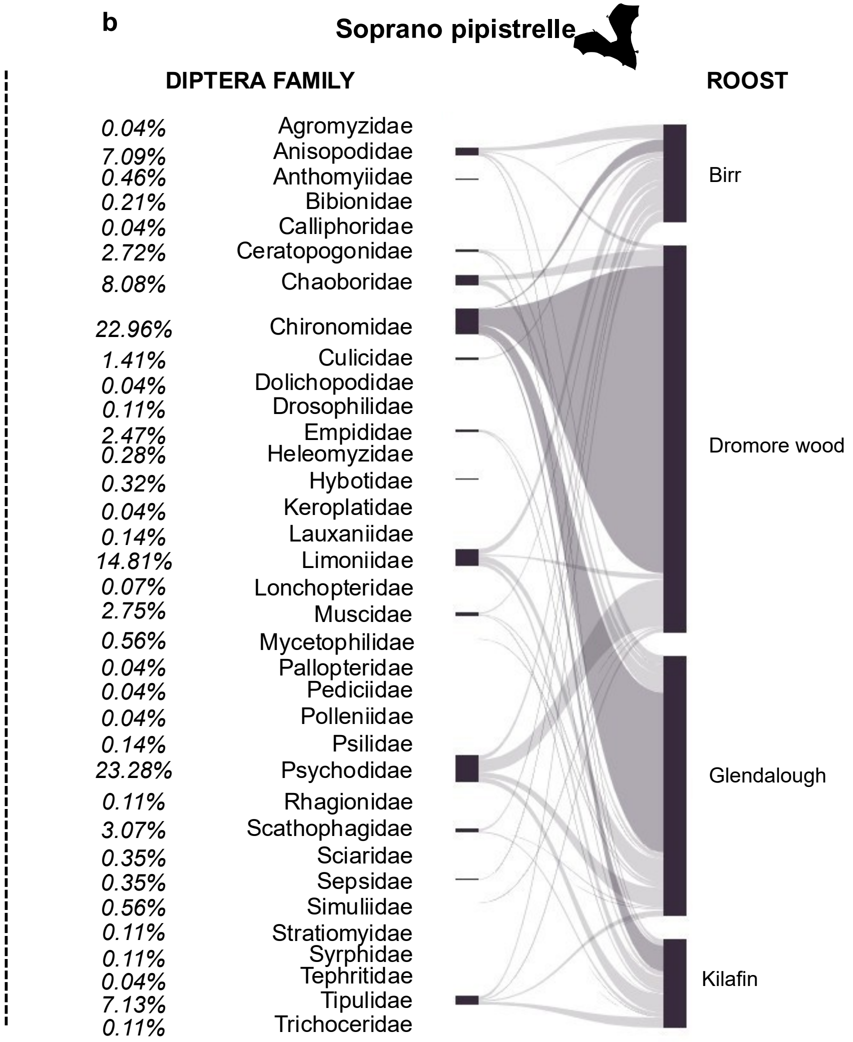

Supplementary Figure S.3B

Supporting Table S3: Details of outliers discarded for the nMDS analysis.

SampleID	Bat species	Roost	Year	Sampling period	nMDS1	nMDS2
D1586_21	Soprano pipistrelle	Glendalough	2023	Gestation	-2879.1	1153.415
D1631_23	Brown long-eared bat	Killmore	2023	Post-lactation	-10.7066	-1.50344
D3_2	Brown long-eared bat	Killmore	2021	Gestation	-2.88731	-2.34257
D1426_13	Brown long-eared bat	Killmore	2022	Post-lactation	-0.34752	-1.21338
D1426_12	Brown long-eared bat	Killmore	2022	Post-lactation	-0.31472	-1.21219
D1118_14	Brown long-eared bat	Glengarriff	2022	Gestation	-0.28079	-1.22061
D1155_5	Soprano pipistrelle	Kilafin	2022	Lactation	-0.02778	-1.21098
D589_14	Brown long-eared bat	Killmore	2021	Post-lactation	-0.27187	-1.2112
D589_16	Brown long-eared bat	Killmore	2021	Post-lactation	-0.26608	-1.2135
D589_6	Brown long-eared bat	Killmore	2021	Post-lactation	-0.26603	-1.2135
D51_31	Brown long-eared bat	Miltown	2021	Gestation	-0.26425	-0.21315
D779_14	Brown long-eared bat	Letterfrack	2021	Post-lactation	3350.001	991.1094
D1625_19	Brown long-eared bat	Letterfrack	2023	Post-lactation	-0.02771	-1.5773

Supporting Information Figure S4

Non-metric multidimensional scaling plot showing the dissimilarity in diet composition across bat species (Top) and Brown long-eared with all samples included (Bottom).

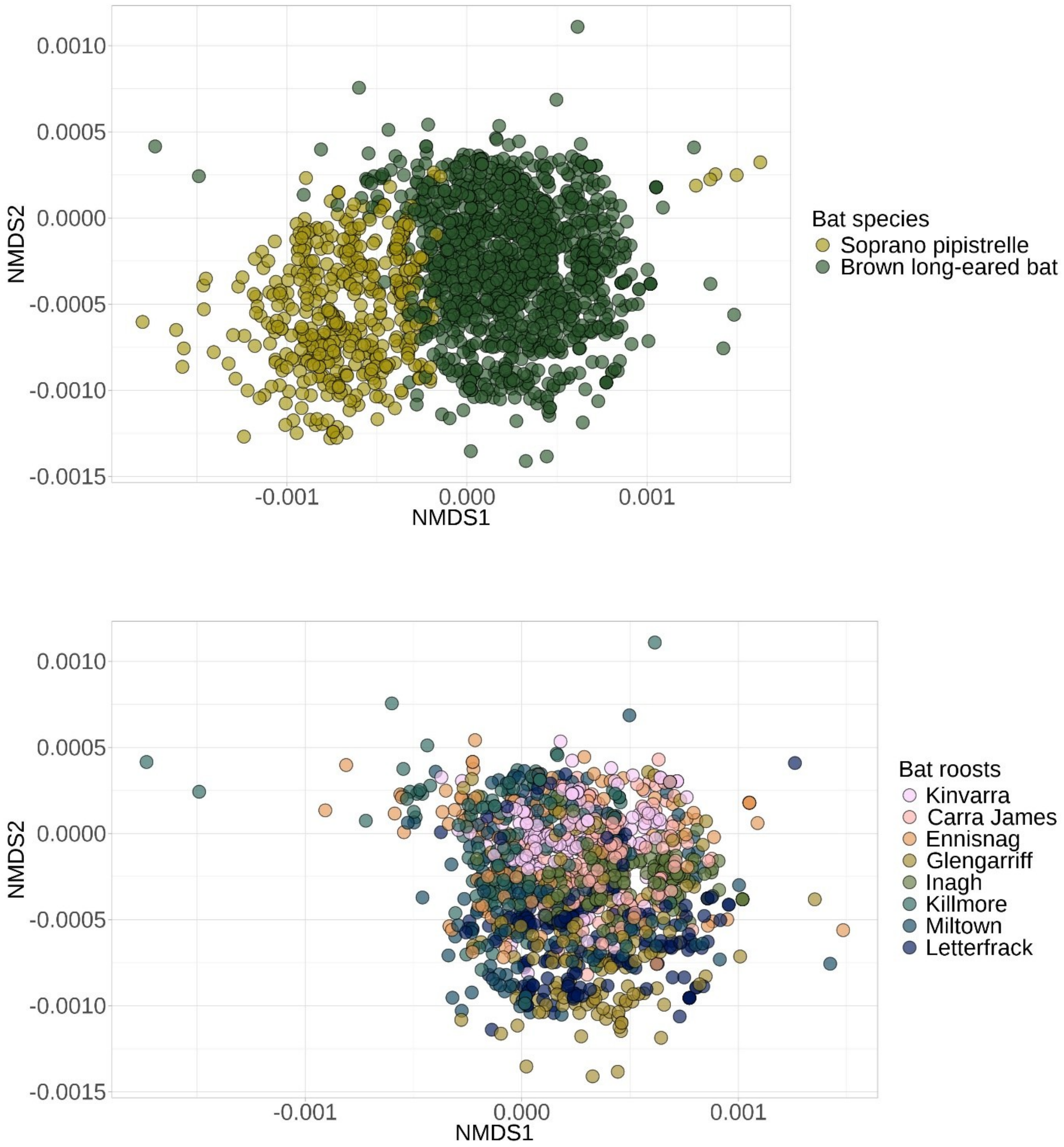

Supplement: Supplementary file 1 — Appendix S1: Supporting Information. [file ECE3-15-e72310-s001.pdf]
